# Income-related inequality in quality-adjusted life expectancy in Korea at the national and district levels

**DOI:** 10.1186/s12955-020-01302-6

**Published:** 2020-02-27

**Authors:** Dohee Lim, Jinwook Bahk, Minsu Ock, Ikhan Kim, Hee-Yeon Kang, Yeon-Yong Kim, Jong Heon Park, Young-Ho Khang

**Affiliations:** 1 Institute of Health Policy and Management, Seoul National University Medical Research Center, Seoul, South Korea; 2 Department of Public Health, Keimyung University, Daegu, Korea, Seoul, South Korea; 3 Department of Preventive Medicine, University of Ulsan College of Medicine, Seoul, South Korea; 4 Department of Health Policy and Management, Seoul National University College of Medicine, Seoul, South Korea; 5 Big Data Steering Department, National Health Insurance Service, Wonju, South Korea

**Keywords:** Income, Life expectancy, Quality of life, Socioeconomic factors, Republic of Korea

## Abstract

**Background:**

The aim of this study was to measure differences in quality-adjusted life expectancy (QALE) by income in Korea at the national and district levels.

**Methods:**

Mortality rates and EuroQol-5D (EQ-5D) scores were obtained from the National Health Information Database of the National Health Insurance Service and the Korea Community Health Survey, respectively. QALE and differences in QALE among income quintiles were calculated using combined 2008–2014 data for 245 districts in Korea. Correlation analyses were conducted to investigate the associations of neighborhood characteristics with QALE and income gaps therein.

**Results:**

QALE showed a graded pattern of inequality according to income, and increased over time for all levels of income and in both sexes, except for low-income quintiles among women, resulting in a widened inequality in QALE among women. In all 245 districts, pro-rich inequalities in QALE were found in both men and women. Districts with higher QALE and smaller income gaps in QALE were concentrated in metropolitan areas, while districts with lower QALE and larger income gaps in QALE were found in rural areas. QALE and differences in QALE by income showed relatively close correlations with socioeconomic characteristics, but relatively weak correlations with health behaviors, except for smoking and indicators related to medical resources.

**Conclusions:**

This study provides evidence of income-based inequalities in health measured by QALE in all subnational areas in Korea. Furthermore, QALE and differences in QALE by income were closely associated with neighborhood-level socioeconomic characteristics.

## Background

Socioeconomic inequalities in life expectancy (LE) have been well documented [1–3]. However, relatively few reports have investigated socioeconomic inequalities in health expectancy (HE). Whereas LE inequalities reflect the differences in mortality experienced by different subgroups of the population, HE inequalities capture the differences in overall health status in terms of both mortality and morbidity [4]. In the current period of rapid aging with longer LE, it is important to identify inequalities in HE, as well as in LE.

At the national level, socioeconomic inequalities in health status are well established, whereas less is known about geographic variations in health status and socioeconomic health inequalities at the subnational level [5]. The health gap in local areas can be exacerbated by various factors related to local social conditions and policies [6], and the interaction of geography with health and health inequality should therefore be a public health concern.

In South Korea (hereafter ‘Korea’), the National Health Information Database (NHID) of the National Health Insurance Service [7] and the Korean Community Health Survey (KCHS) [8] provide information on mortality rates [9] and health-related quality of life (HRQoL) [10], respectively, according to income at both the national and district levels. This data infrastructure in Korea provides a unique opportunity to investigate not only socioeconomic inequalities in HE, but also variations in inequality across subnational districts. Prior studies have examined geographical inequalities in LE in small areas [11–14]. A recent US study presented socioeconomic inequalities in LE at 40 years of age at the county level [1]. The Global Burden of Disease study presented data on HE, but not on HE inequalities, at the subnational level for several countries [15]. To the best of our knowledge, no prior study has examined variations in income-related inequalities in HE at the subnational level.

In this study, we aimed to calculate the quality-adjusted life expectancy (QALE), which is an HE metric, according to income at the national and district levels, and to identify the relationships of neighborhood characteristics with QALE and income gaps therein.

## Methods

### Data

The study was approved by the National Health Insurance Service of Korea (NHIS-2018-1-430) and the Seoul National University Hospital Institutional Review Board (IRB No. E-1810-008-975).

The information on mortality and HRQoL required for QALE calculations was obtained from the NHID and KCHS, respectively, according to gender, income, and district. Both the NHID and KCHS are considered to be good sources for monitoring income-based health outcomes at the district level, as they have district-level population representation and contain information on household income [8, 9]. A total of 342,439,895 subjects and 1,753,476 deaths from the NHID were analyzed to investigate mortality and 1,577,541 participants from the KCHS were examined to evaluate HRQoL (see Supplementary Tables 1, 2 and 3). District-level classifications were based on the 252 administrative districts as of 2014 in Korea. More details on data, study subjects, and the classification of the districts are presented in the Supplementary Methods. In order to guarantee the minimum population needed to calculate the QALE in small areas [16], the 2008–2014 data were combined. A prior statistical paper recommended a minimum population size of 5000 for calculating life expectancy [16]. In our analysis, the pooled population size during 7 years (2008–2014) for districts ranged from 69,913 to 5,477,912. When we calculated life expectancy by sex and income quintiles in each district, the minimum population size was 6508. The district classification was revised to include 245 districts in order to ensure a geographically coherent grouping in consideration of changes in the administrative districts during the time period of the study.

### Income and district-level neighborhood characteristics

Income was classified into five groups by calculating the quintile of the equivalized income considering the number of households by gender and age. District-level neighborhood characteristics included socioeconomic factors (Gini index, social trust, mean height [reflecting childhood socioeconomic status], population change between 2005 and 2015, and area deprivation index), health behaviors (prevalence of current smoking, high-risk drinking, exercise, and overweight), and healthcare factors (the number of hospital beds and doctors per 1000 population). A total of 11 components were used to construct the area deprivation index. Further details on these variables are presented in the Supplementary Methods.

### Health-related quality of life measure

The KCHS contains the EuroQOL five-dimensional (EQ-5D) 3-level questionnaire, which is a self-reported HRQoL tool that consists of five dimensions (mobility, self-care, usual activities, pain/discomfort, and anxiety/depression), each of which is scored with one of three levels of severity (no problems, some or moderate problems, extreme problems). The EQ-5D questionnaire profiles, which contain 243 possible health states, were matched to Korean population-based preference weights for EQ-5D [17], and the EQ-5D health status scores were estimated by gender, 5-year age-specific group, income, and district.

### Statistical analysis

Based on the calculated mortality rates and the EQ-5D scores, LE and QALE were estimated using the Sullivan method. LE was estimated by calendar year, gender, and income level at the national and district levels during 2008–2014, and QALE was estimated by gender and income levels for 2008–2014 at the district level. The formula used for LE and QALE estimates can be found in the Supplementary Methods. Inter-quintile income differences in LE and QALE, rather than the slope index of inequality, were used to measure socioeconomic health inequality in this study, because inter-quintile differences could be more easily understood by the public and local governmental officials. In addition, the correlation coefficients of inter-quintile income differences with the slope index of inequality were 0.980 for LE and 0.976 for QALE (men and women combined). We conducted correlation analyses of each district’s characteristics with district-level QALE and inter-quintile income differences in QALE. SAS version 9.4 (SAS Institute Inc., Cary, NC, USA) was used for the analysis.

## Results

Table 1 shows the national level of LE and QALE by calendar year, gender, and income in Korea between 2008 and 2014. During the study period, the LE of Koreans increased from 79.86 to 82.10 (a 2.2-year increase), while QALE increased from 75.19 to 76.09 (a 0.9-year increase). The increase in LE and QALE was found at all levels of income and in both men and women, except for low-income quintiles (Q1 and Q2) among women, resulting in a widened inequality in QALE. Both LE and QALE showed a graded pattern of inequality according to income, which held true for all calendar years and for both men and women.

**Table 1:** Life expectancy (LE) and quality-adjusted life expectancy (QALE) by income, gender, and year in Korea at the national level

LE							QALE					
Overall		Income Q1 (Lowest)	Income Q2	Income Q3	Income Q4	Income Q5 (Highest)	Overall	Income Q1 (Lowest)	Income Q2	Income Q3	Income Q4	Income Q5 (Highest)
Total												
2008	79.86	75.90	79.98	80.29	81.05	82.67	75.19	70.34	75.02	75.79	76.90	78.55
2009	80.34	76.41	80.43	80.80	81.60	82.96	75.71	70.92	75.67	76.31	77.38	78.91
2010	80.58	76.71	80.62	81.05	81.83	83.15	75.78	71.14	75.56	76.48	77.51	78.83
2011	80.99	77.44	80.87	81.42	82.13	83.54	75.64	71.20	75.30	76.33	77.25	78.69
2012	81.15	77.42	81.10	81.61	82.40	83.77	76.24	71.70	76.02	77.02	77.83	79.28
2013	81.71	77.95	81.60	82.25	82.93	84.38	76.20	71.48	75.93	77.10	77.80	79.42
2014	82.10	78.26	82.07	82.65	83.37	84.68	76.09	71.31	75.34	76.98	77.89	79.40
Men												
2008	76.28	71.60	76.38	76.93	77.62	79.75	72.91	67.09	72.69	73.94	74.88	77.11
2009	76.72	72.13	76.76	77.2	78.25	80.08	73.64	68.09	73.70	74.26	75.59	77.59
2010	76.96	72.46	76.94	77.45	78.37	80.38	73.81	68.39	73.66	74.52	75.78	77.62
2011	77.37	73.13	77.33	77.90	78.61	80.55	73.80	68.44	73.63	74.63	75.68	77.58
2012	77.59	73.25	77.58	78.08	78.95	80.86	74.38	69.12	74.22	75.14	76.17	78.12
2013	78.20	73.78	78.17	78.82	79.56	81.44	74.61	69.19	74.41	75.64	76.49	78.42
2014	78.66	74.19	78.63	79.15	80.14	81.98	74.56	68.97	74.35	75.30	76.69	78.52
Women												
2008	82.99	80.23	83.21	83.1	83.86	84.82	77.25	73.66	77.14	77.48	78.60	79.67
2009	83.46	80.70	83.67	83.85	84.25	85.05	77.55	73.86	77.39	78.16	78.84	79.86
2010	83.68	80.99	83.86	84.03	84.55	85.17	77.56	74.01	77.30	78.12	78.96	79.73
2011	84.07	81.58	84.10	84.30	84.90	85.69	77.30	73.95	77.00	77.80	78.55	79.50
2012	84.19	81.52	84.26	84.54	85.13	85.86	77.94	74.33	77.78	78.64	79.19	80.17
2013	84.69	82.03	84.67	85.05	85.57	86.50	77.65	73.82	77.32	78.34	79.01	80.17
2014	85.02	82.31	85.10	85.54	85.81	86.58	77.47	73.43	77.08	77.96	78.97	79.96

**Fig. 1:**
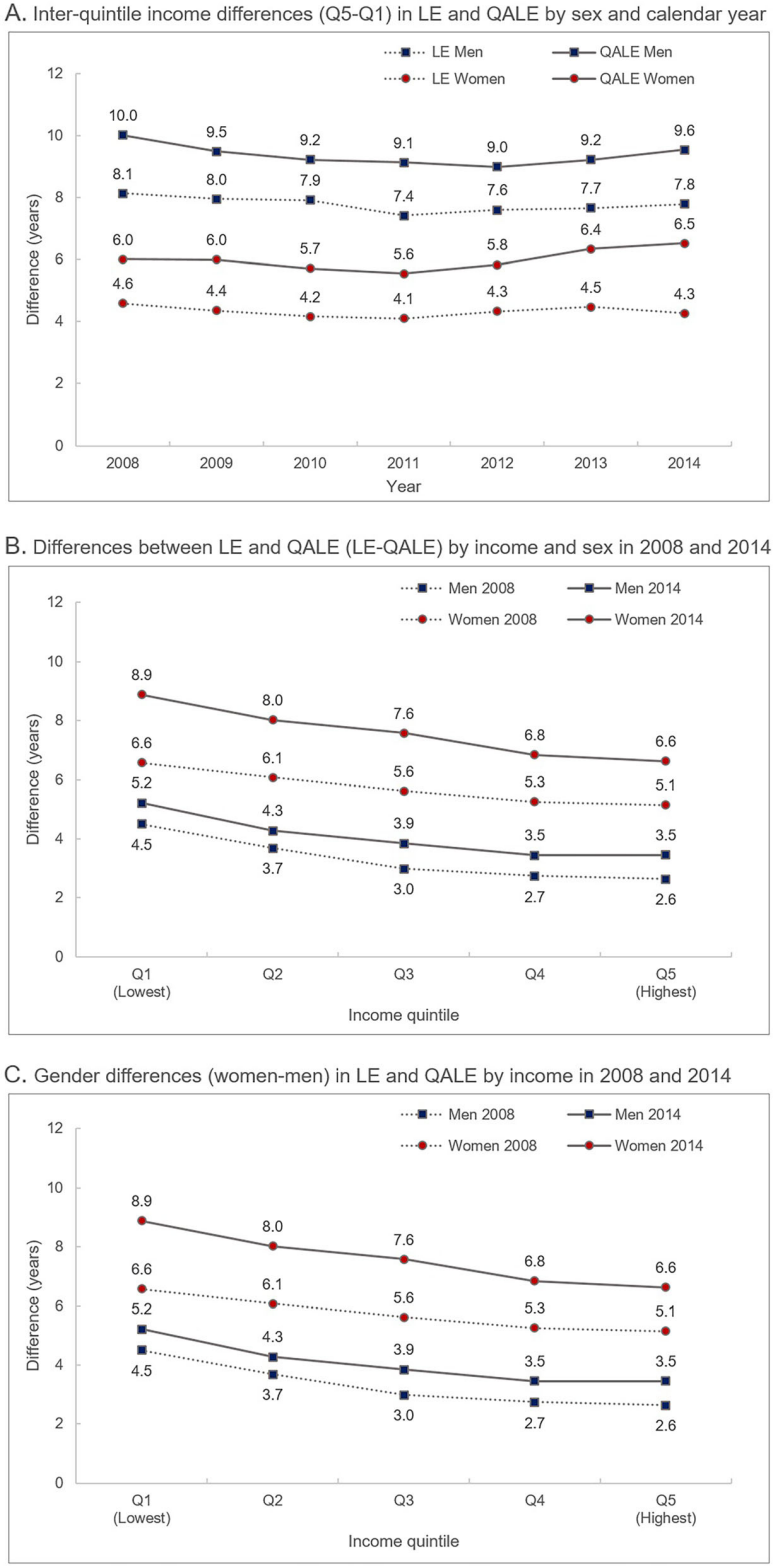
Differences by sex, income, and calendar year in life expectancy (LE) and quality-adjusted life expectancy (QALE)

Figure 1 presents differences in LE and QALE by income, gender, and calendar year. The inter-quintile income difference in QALE was larger than that of LE, and the income gap in QALE and LE was greater for men than for women (Fig. 1-a). Since 2008, the QALE and LE income gaps in men declined, but in women, only the income gap in LE decreased, whereas the inter-quintile income difference in QALE increased (Fig. 1-a). The difference between LE and QALE (LE minus QALE) increased as income became lower, and the magnitude of this difference increased in recent years (Fig. 1-b). The difference between LE and QALE was greater for women than for men due to women’s relative disadvantages in EQ-5D compared to men (Fig. 1-b). Fig. 1-c shows that the gender gap in LE was larger than that in QALE, as is also presented in Fig. 1-b. Moreover, the gender gap in LE and QALE decreased with income, and its magnitude became smaller in recent years (Fig. 1-c). At the district level, the general features of LE and QALE by gender and income level were largely similar to those found at the national level (Supplementary Tables 4, 9 and 10).

**Fig. 2:**
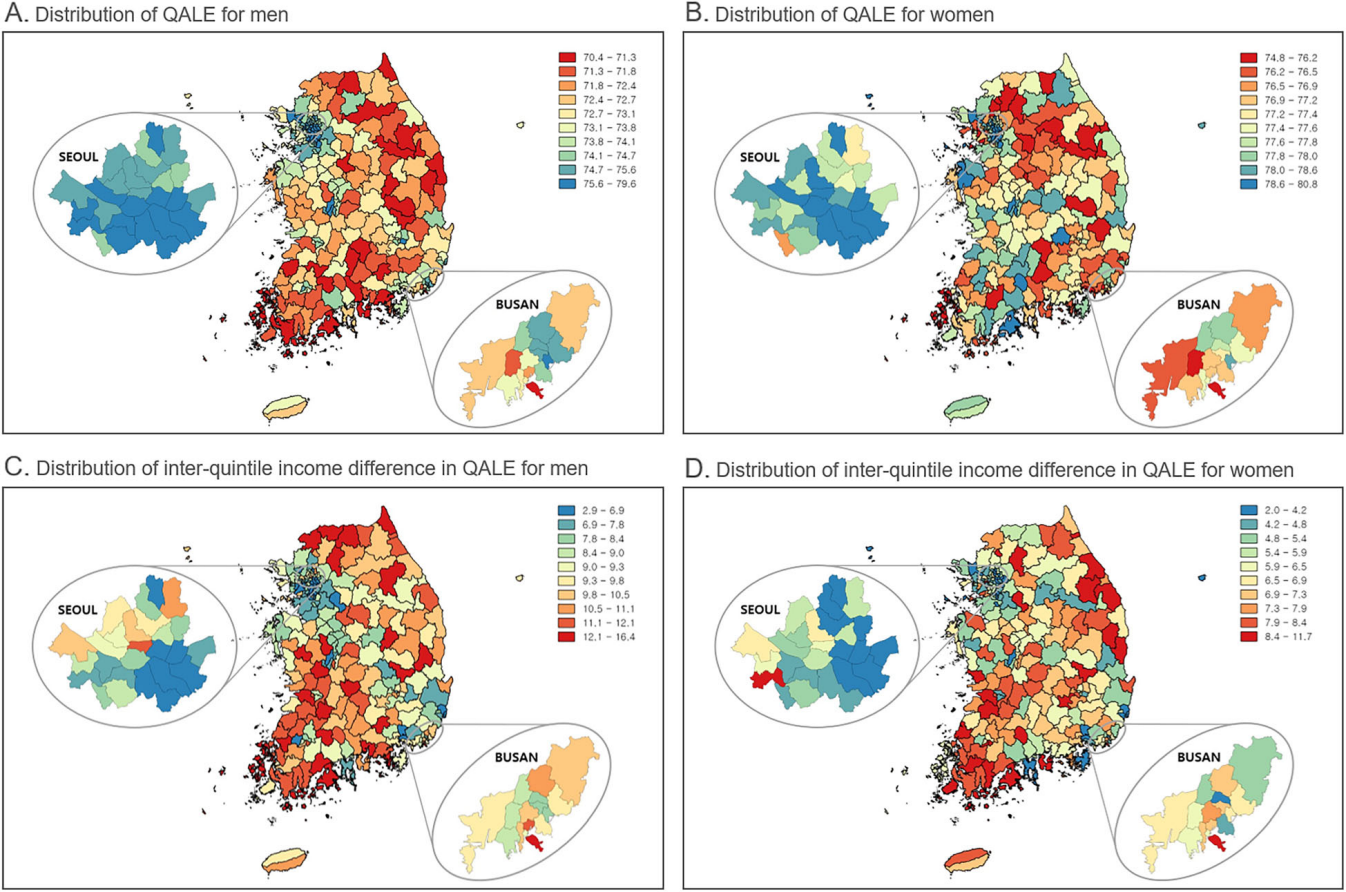
Distribution of district-level quality-adjusted life expectancy (QALE) and differences in QALE among income quintiles by sex in Korea, 2008–2014

Figure 2 shows maps of Korea (including more detailed maps for Seoul and Busan, the two largest mega-cities in Korea) presenting the QALE and inter-quintile income differences in QALE in 245 districts by gender. QALE and the income gap in QALE varied greatly across the 245 districts. In men, QALE was between 70.4 years and 79.6 years (SD = 1.8 years), while in women it was between 74.8 years and 80.8 years (SD = 1.0 year). The corresponding figures for inter-quintile income differences in QALE were between 2.9 years and 16.4 years in men (SD = 2.2 years) and between 2.0 years and 11.7 years in women (SD = 1.8 years). Districts with higher QALE and smaller income gaps in QALE were concentrated in metropolitan areas, especially in Seoul, the capital of Korea, and neighboring areas, while districts with lower QALE and larger income gaps in QALE were found in rural areas (Gangwon and Jeolla Provinces, on the northeast and southwest sides of the Korean peninsula, respectively). At the district level, QALE was negatively correlated with inter-quintile income differences in QALE (see Supplementary Figure 1).

**Fig. 3:**
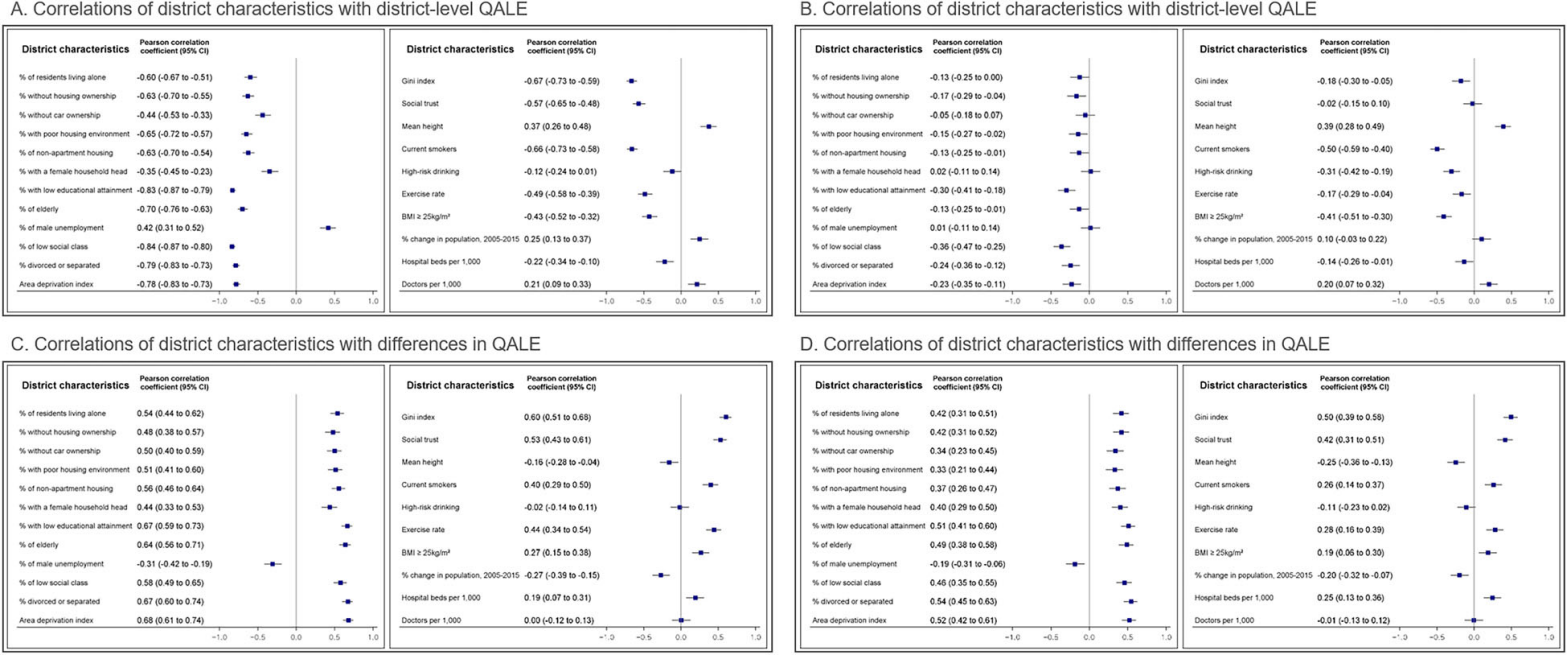
Plots for correlations of district characteristics with district-level quality-adjusted life expectancy (QALE) and differences in QALE among income quintiles in Korea, 2008–2014

Figure 3 shows correlations of district-level neighborhood characteristics with district-level QALE and inter-quintile income differences in QALE. In men, both the QALE (r = − 0.78) and the income gap in QALE (r = 0.68) were closely correlated with the area deprivation index and its components, whereas in women only the income gap in QALE (r = 0.52) showed such an association. This was because men showed a close correlation between the area deprivation index and QALE at all income levels, while women showed a close correlation only at the lowest income level (Supplementary Table 5). Interestingly, unemployment—a component of the area deprivation index—had a positive correlation with QALE and a negative correlation with the income gap in QALE. This was because the level of male unemployment was low in rural areas, where irregular agricultural employment is common. When we separately analyzed the data according to the urbanization level, the magnitude of the correlation coefficient changed, and generally became lower (Supplementary Figures 2 and 3). The inter-quintile income difference in QALE showed a relatively close correlation with indicators of socioeconomic characteristics, such as the Gini index (r = 0.60 for men, r = 0.50 for women). These patterns generally held true for QALE in men, but not for QALE in women, except for the bottom income quintile (Supplementary Table 7). Current smoking prevalence was also closely correlated with QALE (r = − 0.66 for men, r = − 0.50 for women). However, the correlations of QALE and income gaps in QALE with indicators related to health behaviors and medical resources were generally weak. Social trust and exercise prevalence had negative correlations with QALE and positive correlations with the income gap in QALE. This was because the level of social trust and exercise prevalence were high in rural areas. When we separately analyzed the data according to the urbanization level, the correlation coefficients became lower (Supplementary Figures 4 and 5).

## Discussion

This study presented differences in HE by income, as measured by QALE, at both the national and district levels, and confirmed that income-related inequalities in HE existed in both men and women in all 245 districts of Korea. Geographic variations in health inequalities at the subnational level (within-area inequalities) provide a distinct perspective on health inequalities in comparison to geographic variations in health (between-area health inequalities). For example, in this study, the district with the highest HE (the Bundang district) presented an inter-quintile difference in HE of 6.2 years (men and women combined). The HE in the lowest 20% income group of this district was equivalent to the average HE of the 21st district in the HE ranking. Within-district HE inequalities by income provide valuable information for district governments to plan policies and programs to reduce health inequalities in their own districts. Meanwhile, HE and differences in HE by income varied substantially across districts, and these differences were correlated with neighborhood-level socioeconomic characteristics.

To the best of our knowledge, no prior study has examined geographic variations in income-related inequalities with respect to QALE at the subnational level. Chetty and colleagues [1] examined geographic variations in income-related inequalities in LE among US populations, but their analysis did not include any type of HE. Other recent studies examining subnational LE only explored between-area variations in LE [13, 14, 18]. A few studies revealed inequalities in HE according to income at the national level [19]. A recent Korean study presented educational inequalities in QALE and provincial-level variation in QALE, but did not examine geographic variation in socioeconomic inequalities in QALE [20]. In this study, examining income-related inequalities in QALE at the district level was possible because Korea has a good data infrastructure, as represented by NHID (for mortality) and KCHS (for HRQoL), with large sample sizes containing information on income in 245 districts.

The results of this study showed that pro-rich inequalities were more prominent in QALE than in LE. This held true for both men and women, for all years considered, and for all 245 districts (see Supplementary Tables 9 and 10). In 2014, the inter-quintile income difference in LE at the national level was 7.8 years for men and 4.3 years for women, while the inter-quintile income difference in QALE was 9.6 years for men and 6.5 for women. This figure indicates that 81.3% (= 7.8/9.6*100) and 66.2% (= 4.3/6.5*100) of QALE inequalities occurred due to inequalities in LE. This suggests that inequalities in quality of life may play a more important role in creating health inequalities among women than among men.

This study also showed that the extension of LE occurred at the expense of quality of life, which was more prominent in women than in men, and was especially prominent in low-income women. The LE increased by 2.38 years in men and 2.03 in women, respectively, during the past six years between 2008 and 2014, but the QALE increase did not keep pace (1.65 years in men and 0.22 years in women). QALE did not increase in the low-income quintiles of women. A recent projection study indicated that Korean women’s LE is expected to be the highest worldwide, with a 57% likelihood of it surpassing 90 years in 2030 [21]. The results of our study contrast with this optimistic projection, indicating the need for further studies exploring factors leading to the divergence of LE and QALE among Korean women.

This study showed that the correlation between neighborhood characteristics and QALE was somewhat stronger in men than in women. Among the neighborhood characteristics, socioeconomic characteristics (the area deprivation index and Gini index) and current smoking prevalence showed stronger correlations with QALE than healthcare factors. A recent US study similarly showed that county-level LE had closer correlations with socioeconomic and race/ethnicity factors, as well as with behavioral and metabolic risk factors, than with health care factors [12]. A prior study also reported that factors relating to access to health care were not associated with LE at the county level in the US [1]. In this study, income inequality, as measured by the Gini index, was strongly associated with QALE and inequalities in QALE in men, while this was not true for women. The income inequality hypothesis has been debated in terms of potential mechanisms linking income inequality and health [22]. Social capital has been suggested as such a mechanism. However, in this study, social trust, a measure of social capital, was negatively (rather than positively) associated with QALE. This negative correlation occurred because rural areas with sustained high levels of social trust recorded low levels of QALE. Further studies considering the multilevel nature of individual income and neighborhood income inequalities would be warranted to explore the mechanisms and gender differences found in this study.

This study has certain limitations. First, when calculating QALE, the EQ-5D score of the 20- to 24-year-old age group was used for younger ages. This was because the KCHS surveys, which were the source of the EQ-5D data, were only conducted among those aged 19 and over. In practice, information on LE and HE at 0 years old, which is a summary of the entire lifespan, is more useful for planning and evaluating health policies. Second, spatial autocorrelation was not considered in the analysis. This choice was made to allow local government officials and local health departments to utilize statistical findings from the data obtained from their own districts. Third, the magnitude of the relationships of LE and HE with income or district area should not be interpreted as causal effects of having more money or living in those specific districts. This study was conducted to describe the magnitude of these associations, rather than to explore causal effects.

## Conclusion

This study revealed the existence of income-based inequalities in health measured by QALE in all subnational areas in Korea, and showed close associations of the magnitude of QALE and income gaps therein with neighborhood socioeconomic characteristics.

## Supplementary information

**Additional file 1 **Supplementary methods. Supplementary **Table 1.** Number of subjects from the National Health Information Database of National Health Insurance Service by year and income quintile. Supplementary **Table 2.** Number of deaths from the National Health Information Database of the National Health Insurance Service by year and income quintile. Supplementary **Table 3**. Number of subjects from the Korean Community Health Survey by year and income quintile. Supplementary **Table 4**. Central tendency (mean and median) and dispersion (standard deviation = SD, range, and interquartile range = IQR) for district-level quality-adjusted life expectancy (QALE) and life expectancy (LE) by income quintile, 2008-2014. Supplementary **Table 5.** Correlations of the area deprivation index with district-level quality-adjusted life expectancy (QALE) by gender and income quintile, 2008-2014. Supplementary **Table 6.** Correlations of the area deprivation index with district-level life expectancy (LE) by gender and income quintile, 2008-2014. Supplementary **Table 7.** Correlations of district characteristics with district-level quality-adjusted life expectancy (QALE) by gender and income quintile, 2008-2014. Supplementary **Table 8.** Correlations of district characteristics with district-level life expectancy (LE) by gender and income quintile, 2008-2014. Supplementary **Figure 1.** Correlations of quality-adjusted life expectancy (QALE) with inter-quintile income differences in QALE at the district level. Supplementary **Figure 2.** Plots of correlations of the area deprivation index with district-level quality-adjusted life expectancy (QALE) by gender and urbanization level. Supplementary **Figure 3.** Plots of correlations of the area deprivation index with inter-quintile income differences in district-level quality-adjusted life expectancy (QALE) by gender and urbanization level. Supplementary **Figure 4.** Plots of correlations of district characteristics with district-level quality-adjusted life expectancy by gender and urbanization level. Supplementary **Figure 5.** Plots of correlations of district characteristics with inter-quintile income differences in district-level quality-adjusted life expectancy by gender and urbanization level.

**Additional file 2 **: Supplementary **Table 9.** Quality-adjusted life expectancy (QALE) by income and gender among 245 districts in Korea, 2008-2014. Supplementary **Table 10.** Life expectancy (LE) by income and gender among 245 districts in Korea, 2008-2014. Supplementary **Table 11.** Distribution of population size and the number of death by income and gender among 245 districts in Korea, 2008-2014. Supplementary **Table 12.** Mean and 95% CI of EQ-5D scores by income and gender in Korea, 2008-2014. Supplementary **Table 13.** Distribution of EQ-5D scores by income and gender among 245 districts in Korea, 2008-2014.

## Data Availability

The data that support the findings of this study are available from National Health Insurance Sharing Service (https://nhiss.nhis.or.kr) but restrictions apply to the availability of these data, which were used under license for the current study, and so are not publicly available.

## Abbreviations

95% CI: 95% confidence interval
EQ-5D: EuroQol five-dimensional
HE: Health expectancy
HRQoL: Health related quality of life
KCHS: Korean Community Health Survey
LE: Life expectancy
NHID: National Health Information Database
QALE: Quality-adjusted life expectancy
