# Supplementary material for: Income-related inequality in quality-adjusted life expectancy in Korea at the national and district levels
Source: Health Qual Life Outcomes. 2020 Feb 27;18:45. doi: 10.1186/s12955-020-01302-6 (PMC7045490; doi:10.1186/s12955-020-01302-6)
Supplement: Supplementary file 1 — Additional file 1 Supplementary methods. Supplementary Table 1. Number of subjects from the National Health Information Database of National Health Insurance Service by year and income quintile. Supplementary Table 2. Number of deaths from the National Health Information Database of the National Health Insurance Service by year and income quintile. Supplementary Table 3. Number of subjects from the Korean Community Health Survey by year and income quintile. Supplementary Table 4. Central tendency (mean and median) and dispersion (standard deviation = SD, range, and interquartile range = IQR) for district-level quality-adjusted life expectancy (QALE) and life expectancy (LE) by income quintile, 2008-2014. Supplementary Table 5. Correlations of the area deprivation index with district-level quality-adjusted life expectancy (QALE) by gender and income quintile, 2008-2014. Supplementary Table 6. Correlations of the area deprivation index with district-level life expectancy (LE) by gender and income quintile, 2008-2014. Supplementary Table 7. Correlations of district characteristics with district-level quality-adjusted life expectancy (QALE) by gender and income quintile, 2008-2014. Supplementary Table 8. Correlations of district characteristics with district-level life expectancy (LE) by gender and income quintile, 2008-2014. Supplementary Figure 1. Correlations of quality-adjusted life expectancy (QALE) with inter-quintile income differences in QALE at the district level. Supplementary Figure 2. Plots of correlations of the area deprivation index with district-level quality-adjusted life expectancy (QALE) by gender and urbanization level. Supplementary Figure 3. Plots of correlations of the area deprivation index with inter-quintile income differences in district-level quality-adjusted life expectancy (QALE) by gender and urbanization level. Supplementary Figure 4. Plots of correlations of district characteristics with district-level quality-adjusted life expectancy by [file 12955_2020_1302_MOESM1_ESM.docx]

**Supplementary methods**

**Data and study subjects**

This study employed several data sources. First, to estimate mortality by income level for the period 2008-2014, we used the eligibility database of the National Health Information Database (NHID) linked to death certificate data from Statistics Korea. The NHID is a database provided by the National Health Insurance Service (NHIS), a compulsory health insurance scheme covering the whole Korean population, and is considered to be a good source for monitoring mortality and life expectancy (LE) across national, provincial, and municipal levels with representation of the entire Korean population [1]. Information on mortality rates was obtained by linking the NHID eligibility database to mortality registries for the period 2008-2014. Eayres and Williams recommended a population size of over 5,000 as a reasonable minimum for estimating life expectancy in small areas. We integrated data from 2008 to 2014 to ensure stable mortality rates at the district level [2]. From this, aggregate data for the numbers of population and deaths were derived according to gender, 5-year age-specific groups (<1, 1-4, 5-9, 10-14, …, and ≥85 years), income quintiles, and 245 districts. The analysis was conducted among all health insurance subscribers in Korea, and excluded foreigners and soldiers. A total of 342,439,895 subjects (171,287,729 men, 171,152,166 women) and 1,753,476 deaths (970,928 men, 782,548 women) were used for the analysis (Supplementary Table 1-2).

Secondly, to estimate health-related quality of life (HRQoL) by gender, age, income, and district, we used the Korea Community Health Survey (KCHS). The KCHS was launched as a nationwide, community-based, cross-sectional survey in 2008 to produce comparable health statistics at the district level, on an annual basis, with approximately 900 participants aged 19 years or older in each district in Korea. Other details about the KCHS have been described elsewhere [3].

In the KCHS, information on health is obtained using the EuroQOL five-dimensional (EQ-5D) 3-level questionnaire, which is a self-reported health-related quality of life tool that consists of five dimensions (mobility, self-care, usual activities, pain/discomfort, and anxiety/depression) each of which is scored with one of three levels of severity (no problems, some or moderate problems, or extreme problems). The EQ-5D questionnaire profiles, which have 243 possible health states, were matched to Korean population-based preference weights for the EQ-5D using a representative sample (1,037 people) of the Korean population using the time trade-off method [4], and EQ-5D health status scores were estimated by gender, 5-year age-specific group (20-24, 25-29, …, and ≥ 85 years), income quintile, and district (among 245 districts). Since the KCHS is a survey of people aged 19 and over, for those younger than 20 years, the EQ-5D health status score of the 20- to 24-year-old age group was used instead. For our analyses, a total of 1,577,541 respondents over 20 years of age from 2008 to 2014 were used, except for respondents who had missing information for EQ-5D questions and household size (Supplementary Table 3). The data between 2008 and 2014 were integrated to ensure a stable EQ-5D health status score estimation [2].

Based on the calculated mortality rates and the EQ-5D health status scores, LE and QALE were estimated using the Sullivan method. LE was estimated by calendar year, gender, and income level at the national and district levels during 2008-2014, and QALE was estimated by gender and income level for 2008-2014 at the district level. The formulas used for the LE and QALE estimates are as follows.

$$\boldsymbol{e}_{\boldsymbol{x}}\text{ (Life expectancy)}\boldsymbol{=}\frac{\boldsymbol{T}_{\boldsymbol{x}} \text{(Total number of years lived from x)}}{\boldsymbol{l}_{\boldsymbol{x}} \text{(Number sur}\text{viving}\text{ to age x)}}$$

$$\boldsymbol{He}_{\boldsymbol{x}}\text{ (Health expectancy)}\boldsymbol{=}\frac{\sum_{\boldsymbol{i=x}}^{\boldsymbol{\omega}} \boldsymbol{(}\boldsymbol{L}_{\boldsymbol{i}}\boldsymbol{\times}\text{ }\pi_{i}\text{)}}{\boldsymbol{l}_{\boldsymbol{x}} \text{(Number surviving to age x)}}$$

$$\text{(}L_{i}=Person-years lived at age i, \pi_{i}=\text{EQ-5D health status score at age i})$$

**Income variables**

From the NHID eligibility database, we obtained information on national health insurance premiums at the end of the year (December 31) as a proxy for income levels. When the year-end health insurance premium was not available, the most recent national health insurance premium was used instead. The usefulness of the national health insurance premium as a proxy for income levels in monitoring the income gap in health outcomes has been demonstrated in previous studies [5, 6]. We calculated the equivalized health insurance premium to take into account household size using the following equation.

$$\text{Equivalized}\text{ annual household income}=\frac{\text{(Annual household income)}}{{(\text{Number of household members})}^{0.5}}$$

In KCHS, income data were calculated from the self-reported monthly or yearly household income information, which was collected as a continuous variable in 2008-2013 and as a categorical variable in 2014. The categorized monthly household income in 2014 was converted to a continuous variable by taking the median value of each category. More details about the income data in the KCHS have been described elsewhere [7]. Since the KCHS data were integrated in 2008-2014, household income was adjusted during the survey period using the consumer price index for 2010, considering fluctuations in inflation during 2008-2014. For 110 579 (7.0%) respondents who had missing information on household income, the values were replaced by imputation, and the age, gender, family members, occupation, and educational attainment of the respondents were used to estimate the missing value.

**Administrative districts**

The administrative districts of the KCHS were classified into 252 districts according to the government administrative classification in 2014. However, taking into account changes in administrative units from 2008 to 2014, the administrative districts were reclassified to include 245 districts, in order to maintain consistency in the geographic classification during the study period. The 245 districts were categorized as metropolitan, urban and rural areas according to the urbanization level. More details about the reclassification of the districts have been presented elsewhere [7]. Further information on provinces and urbanity in each district are presented in Supplementary Table 9.

**District-level neighborhood characteristics**

District-level neighborhood characteristics were examined in terms of socioeconomic characteristics, health behaviors, and indicators related to medical resources. All indicators for these neighborhood characteristics were calculated based on the direct standardization method, using the 2010 population as the standard population.

*Socioeconomic characteristics of districts*

The variables for the socioeconomic characteristic of districts took into account the following: the Gini index, which indicates the degree of income inequality in the region; social trust; average height, which represents childhood socioeconomic status; the population change rate between 2005 and 2015; and the area deprivation index.

In total, 11 variables were used to construct the area deprivation index: the proportion of residents living alone, the proportion of households without housing ownership, the proportion of households without car ownership, the proportion of households with a poor housing environment, the proportion of households with living in non-apartment housing, the proportion of households with a female household head, the proportion of people aged 30-64 with no high school diploma, the proportion of the population aged 65 or over, the proportion of male unemployment, the proportion of individuals with a low social class, and the proportion of separated, divorced, or widowed individuals among people aged 15 years and over. Each variable was standardized using the Z-score and then combined to calculate the area deprivation index [8].

The information on social trust and population height was obtained from the KCHS, and the remaining variables on socioeconomic characteristics came from the Korean Statistical Information Service (KOSIS). In the KCHS, the level of social trust was surveyed twice, in 2011 and 2013, and the proportion of people who answered “yes” to the question “People in our neighborhood can trust each other” was used as the level of social trust of the district.

*Health behaviors at the district level*

Health behaviors included current smoking prevalence, high-risk drinking prevalence, exercise prevalence, and overweight prevalence (body mass index (BMI) ≥ 25 kg/m^2^). The variables for these health behaviors were obtained from the KCHS. The prevalence of high-risk drinking was calculated by the proportion of individuals who drank more than 7 drinks (for men) and 5 drinks (for women) at one sitting in the most recent year at least twice per week. Exercise prevalence was calculated as the proportion of individuals who engaged in intense physical activity for more than 20 minutes a day for more than 3 days a week or moderate physical activity for more than 30 minutes a day for more than 5 days a week for the past week.

*Indicators related to medical resources*

Regarding medical resources, the numbers of hospital beds and doctors per 1,000 population were considered. Average values of the data from KOSIS for 2008-2014 were used [9, 10].

**Supplementary References**

1. Bahk J, Kim YY, Kang HY, et al. Using the national health information database of the national health insurance service in Korea for monitoring mortality and life expectancy at national and local levels. J Korean Med Sci. 2017;32:1764-70.
2. Eayres D, Williams ES. Evaluation of methodologies for small area life expectancy estimation. J Epidemiol Community Health. 2004;58:243-9.
3. Kang YW, Ko YS, Kim YJ, et al. Korea community health survey data profiles. Osong Public Health Res Perspect. 2015;6:211-7.
4. Lee YK, Nam HS, Chuang LH, et al. South Korean time trade-off values for EQ-5D health states: modeling with observed values for 101 health states. Value Health. 2009;12:1187-93.
5. Khang YH, Bahk J, Yi N, Yun SC. Age- and cause-specific contributions to income difference in life expectancy at birth: findings from nationally representative data on one million South Koreans. Eur J Public Health. 2016;26:242-8.
6. Khang YH, Yang S, Cho HJ, Jung-Choi K, Yun SC. Decomposition of socio-economic differences in life expectancy at birth by age and cause of death among 4 million South Korean public servants and their dependents. Int J Epidemiol. 2010;39:1656-66.
7. Kim I, Bahk J, Yoon TH, Yun SC, Khang YH. Income differences in smoking prevalences in 245 districts of South Korea: patterns by area deprivation and urbanity, 2008-2014. J Prev Med Public Health. 2017;50:100-26.
8. Yoon TH, Noh M, Han J, Jung-Choi K, Khang YH. Deprivation and suicide mortality across 424 neighborhoods in Seoul, South Korea: a Bayesian spatial analysis. Int J Public Health. 2015;60:969-76.
9. Korean Statistical Information Service. The number of medical institution beds per 1,000 population [Internet]. Daejeon: Korean Statistical Information Service; 2017 [cited 2018 Sep 30] Available from: <http://kosis.kr/statHtml/statHtml.do?orgId=101&tblId=DT_1YL20971&conn_path=I3>.
10. Korean Statistical Information Service. The number of doctors engaged in medical institutions per 1,000 population [Internet]. Daejeon: Korean Statistical Information Service; 2017 [cited 2018 Sep 30] Available from: <http://kosis.kr/statHtml/statHtml.do?orgId=101&tblId=DT_1YL20981&conn_path=I3>.

**Supplementary Table 1. Number of subjects from the National Health Information Database of National Health Insurance Service by year and income quintile**

|  | Overall | Income Q1 (Lowest) | Income Q2 | Income Q3 | Income Q4 | Income Q5 (Highest) |
| --- | --- | --- | --- | --- | --- | --- |
| Total |  |  |  |  |  |  |
| 2008-14 | 342,439,895 | 68,738,002 | 68,316,489 | 68,453,278 | 68,455,558 | 68,476,568 |
| 2008 | 48,383,334 | 9,703,385 | 9,664,548 | 9,666,363 | 9,673,925 | 9,675,113 |
| 2009 | 48,647,975 | 9,770,578 | 9,695,137 | 9,732,836 | 9,720,945 | 9,728,479 |
| 2010 | 48,035,240 | 9,632,253 | 9,590,744 | 9,602,814 | 9,605,039 | 9,604,390 |
| 2011 | 49,086,809 | 9,883,028 | 9,762,013 | 9,813,201 | 9,812,676 | 9,815,891 |
| 2012 | 49,277,624 | 9,885,518 | 9,838,410 | 9,847,371 | 9,852,993 | 9,853,332 |
| 2013 | 49,467,637 | 9,918,012 | 9,877,287 | 9,888,796 | 9,891,641 | 9,891,901 |
| 2014 | 49,541,276 | 9,945,228 | 9,888,350 | 9,901,897 | 9,898,339 | 9,907,462 |
| Men |  |  |  |  |  |  |
| 2008-14 | 171,287,729 | 34,360,027 | 34,195,938 | 34,246,653 | 34,234,472 | 34,250,639 |
| 2008 | 24,237,161 | 4,852,502 | 4,844,862 | 4,845,890 | 4,847,362 | 4,846,545 |
| 2009 | 24,359,235 | 4,879,243 | 4,869,949 | 4,872,762 | 4,866,074 | 4,871,207 |
| 2010 | 24,040,175 | 4,821,211 | 4,800,528 | 4,803,765 | 4,808,698 | 4,805,973 |
| 2011 | 24,557,434 | 4,942,446 | 4,885,966 | 4,911,589 | 4,906,773 | 4,910,660 |
| 2012 | 24,635,159 | 4,939,352 | 4,925,195 | 4,920,211 | 4,924,125 | 4,926,276 |
| 2013 | 24,717,446 | 4,951,207 | 4,939,844 | 4,941,040 | 4,943,193 | 4,942,162 |
| 2014 | 24,741,119 | 4,974,066 | 4,929,594 | 4,951,396 | 4,938,247 | 4,947,816 |
| Women |  |  |  |  |  |  |
| 2008-14 | 171,152,166 | 34,377,975 | 34,120,551 | 34,206,625 | 34,221,086 | 34,225,929 |
| 2008 | 24,146,173 | 4,850,883 | 4,819,686 | 4,820,473 | 4,826,563 | 4,828,568 |
| 2009 | 24,288,740 | 4,891,335 | 4,825,188 | 4,860,074 | 4,854,871 | 4,857,272 |
| 2010 | 23,995,065 | 4,811,042 | 4,790,216 | 4,799,049 | 4,796,341 | 4,798,417 |
| 2011 | 24,529,375 | 4,940,582 | 4,876,047 | 4,901,612 | 4,905,903 | 4,905,231 |
| 2012 | 24,642,465 | 4,946,166 | 4,913,215 | 4,927,160 | 4,928,868 | 4,927,056 |
| 2013 | 24,750,191 | 4,966,805 | 4,937,443 | 4,947,756 | 4,948,448 | 4,949,739 |
| 2014 | 24,800,157 | 4,971,162 | 4,958,756 | 4,950,501 | 4,960,092 | 4,959,646 |

**Supplementary Table 2. Number of deaths from the National Health Information Database of the National Health Insurance Service by year and income quintile**

|  | Overall | Income Q1 (Lowest) | Income Q2 | Income Q3 | Income Q4 | Income Q5 (Highest) |
| --- | --- | --- | --- | --- | --- | --- |
| Total |  |  |  |  |  |  |
| 2008-14 | 1,753,476 | 494,272 | 339,938 | 336,183 | 314,344 | 268,739 |
| 2008 | 239,264 | 67,953 | 46,070 | 45,780 | 42,982 | 36,479 |
| 2009 | 240,172 | 67,865 | 46,528 | 46,361 | 42,719 | 36,699 |
| 2010 | 245,488 | 68,467 | 48,003 | 47,197 | 44,072 | 37,749 |
| 2011 | 250,327 | 70,456 | 48,257 | 47,925 | 45,072 | 38,617 |
| 2012 | 259,929 | 73,154 | 50,460 | 50,021 | 46,359 | 39,935 |
| 2013 | 258,702 | 73,090 | 50,234 | 49,303 | 46,447 | 39,628 |
| 2014 | 259,594 | 73,287 | 50,386 | 49,596 | 46,693 | 39,632 |
| Men |  |  |  |  |  |  |
| 2008-14 | 970,928 | 285,206 | 192,698 | 184,950 | 170,377 | 137,697 |
| 2008 | 133,163 | 39,568 | 26,068 | 25,154 | 23,472 | 18,901 |
| 2009 | 133,947 | 39,585 | 26,601 | 25,703 | 23,183 | 18,875 |
| 2010 | 136,586 | 39,905 | 27,223 | 26,141 | 23,903 | 19,414 |
| 2011 | 139,232 | 40,339 | 27,762 | 26,562 | 24,597 | 19,972 |
| 2012 | 143,148 | 41,770 | 28,515 | 27,247 | 25,219 | 20,397 |
| 2013 | 142,187 | 41,761 | 28,336 | 26,959 | 24,926 | 20,205 |
| 2014 | 142,665 | 42,278 | 28,193 | 27,184 | 25,077 | 19,933 |
| Women |  |  |  |  |  |  |
| 2008-14 | 782,548 | 209,066 | 147,240 | 151,233 | 143,967 | 131,042 |
| 2008 | 106,101 | 28,385 | 20,002 | 20,626 | 19,510 | 17,578 |
| 2009 | 106,225 | 28,280 | 19,927 | 20,658 | 19,536 | 17,824 |
| 2010 | 108,902 | 28,562 | 20,780 | 21,056 | 20,169 | 18,335 |
| 2011 | 111,095 | 30,117 | 20,495 | 21,363 | 20,475 | 18,645 |
| 2012 | 116,781 | 31,384 | 21,945 | 22,774 | 21,140 | 19,538 |
| 2013 | 116,515 | 31,329 | 21,898 | 22,344 | 21,521 | 19,423 |
| 2014 | 116,929 | 31,009 | 22,193 | 22,412 | 21,616 | 19,699 |

**Supplementary Table 3. Number of subjects from the Korean Community Health Survey by year and income quintile**

|  | Overall | Income Q1 (Lowest) | Income Q2 | Income Q3 | Income Q4 | Income Q5 (Highest) |
| --- | --- | --- | --- | --- | --- | --- |
| Total |  |  |  |  |  |  |
| 2008-14 | 1,577,541 | 312,154 | 315,144 | 322,561 | 314,076 | 313,606 |
| 2008 | 217,955 | 43,038 | 44,156 | 44,634 | 42,735 | 43,392 |
| 2009 | 228,240 | 45,928 | 45,027 | 45,133 | 46,134 | 46,018 |
| 2010 | 226,619 | 46,141 | 45,469 | 44,549 | 44,570 | 45,890 |
| 2011 | 226,495 | 44,917 | 45,809 | 44,736 | 47,102 | 43,931 |
| 2012 | 226,248 | 44,707 | 47,108 | 44,039 | 45,300 | 45,094 |
| 2013 | 226,045 | 45,486 | 43,803 | 47,795 | 43,327 | 45,634 |
| 2014 | 225,939 | 41,937 | 43,772 | 51,675 | 44,908 | 43,647 |
| Men |  |  |  |  |  |  |
| 2008-14 | 716,967 | 142,812 | 140,997 | 147,318 | 141,551 | 144,289 |
| 2008 | 100,344 | 20,071 | 21,084 | 19,061 | 19,944 | 20,184 |
| 2009 | 105,970 | 20,953 | 21,568 | 20,141 | 21,885 | 21,423 |
| 2010 | 103,443 | 20,695 | 20,574 | 20,779 | 20,212 | 21,183 |
| 2011 | 101,798 | 20,135 | 20,380 | 20,244 | 21,480 | 19,559 |
| 2012 | 101,725 | 19,963 | 20,827 | 20,285 | 20,301 | 20,349 |
| 2013 | 101,453 | 20,563 | 20,318 | 21,462 | 18,362 | 20,748 |
| 2014 | 102,234 | 20,432 | 16,246 | 25,346 | 19,367 | 20,843 |
| Women |  |  |  |  |  |  |
| 2008-14 | 860,574 | 169,342 | 174,147 | 175,243 | 172,525 | 169,317 |
| 2008 | 117,611 | 22,967 | 23,072 | 25,573 | 22,791 | 23,208 |
| 2009 | 122,270 | 24,975 | 23,459 | 24,992 | 24,249 | 24,595 |
| 2010 | 123,176 | 25,446 | 24,895 | 23,770 | 24,358 | 24,707 |
| 2011 | 124,697 | 24,782 | 25,429 | 24,492 | 25,622 | 24,372 |
| 2012 | 124,523 | 24,744 | 26,281 | 23,754 | 24,999 | 24,745 |
| 2013 | 124,592 | 24,923 | 23,485 | 26,333 | 24,965 | 24,886 |
| 2014 | 123,705 | 21,505 | 27,526 | 26,329 | 25,541 | 22,804 |

**Supplementary Table 4. Central tendency (mean and median) and dispersion (standard deviation = SD, range, and interquartile range = IQR) for district-level quality-adjusted life expectancy (QALE) and life expectancy (LE) by income quintile, 2008-2014**

|  | QALE (2008-2014) | | | | | |  | LE (2008-2014) | | | | | |
| --- | --- | --- | --- | --- | --- | --- | --- | --- | --- | --- | --- | --- | --- |
|  | Mean | SD | Min | Median | Max | IQR |  | Mean | SD | Min | Median | Max | IQR |
| Total |  |  |  |  |  |  |  |  |  |  |  |  |  |
| Overall | 75.41 | 1.30 | 72.68 | 75.26 | 80.44 | 1.99 |  | 80.43 | 1.31 | 78.18 | 80.17 | 85.88 | 2.03 |
| Income Q1 | 70.34 | 2.31 | 65.24 | 69.97 | 77.57 | 4.05 |  | 76.18 | 2.42 | 70.86 | 75.90 | 84.55 | 4.17 |
| Income Q2 | 75.36 | 1.27 | 71.97 | 75.27 | 79.89 | 1.94 |  | 80.63 | 1.11 | 78.28 | 80.49 | 84.41 | 1.73 |
| Income Q3 | 76.34 | 1.18 | 72.72 | 76.30 | 80.94 | 1.78 |  | 81.19 | 1.12 | 78.87 | 81.03 | 86.51 | 1.77 |
| Income Q4 | 77.25 | 1.24 | 74.12 | 77.14 | 82.09 | 1.77 |  | 81.90 | 1.17 | 79.08 | 81.77 | 86.45 | 1.64 |
| Income Q5 | 78.56 | 1.24 | 75.14 | 78.47 | 83.49 | 1.76 |  | 83.06 | 1.28 | 79.96 | 82.86 | 88.38 | 1.94 |
| Q5-Q1 | 8.23 | 1.82 | 3.73 | 8.30 | 13.03 | 3.15 |  | 6.88 | 1.79 | 2.60 | 6.80 | 11.32 | 3.04 |
| Men |  |  |  |  |  |  |  |  |  |  |  |  |  |
| Overall | 73.35 | 1.76 | 70.42 | 73.06 | 79.64 | 2.99 |  | 76.66 | 1.72 | 73.41 | 76.41 | 82.60 | 2.90 |
| Income Q1 | 67.53 | 2.91 | 60.88 | 67.46 | 76.98 | 4.90 |  | 71.78 | 2.89 | 64.09 | 71.66 | 80.92 | 4.97 |
| Income Q2 | 73.35 | 1.66 | 69.23 | 73.18 | 79.58 | 2.56 |  | 76.93 | 1.54 | 73.33 | 76.95 | 81.61 | 2.45 |
| Income Q3 | 74.35 | 1.59 | 70.35 | 74.28 | 80.25 | 2.51 |  | 77.56 | 1.44 | 73.76 | 77.54 | 83.44 | 2.19 |
| Income Q4 | 75.41 | 1.54 | 70.97 | 75.31 | 80.65 | 2.32 |  | 78.36 | 1.50 | 74.56 | 78.24 | 84.35 | 2.22 |
| Income Q5 | 77.01 | 1.67 | 72.96 | 76.80 | 83.09 | 2.72 |  | 79.88 | 1.65 | 75.90 | 79.71 | 85.42 | 2.73 |
| Q5-Q1 | 9.48 | 2.17 | 2.91 | 9.33 | 16.38 | 3.36 |  | 8.10 | 1.94 | 3.37 | 7.78 | 15.84 | 3.00 |
| Women |  |  |  |  |  |  |  |  |  |  |  |  |  |
| Overall | 77.39 | 1.00 | 74.75 | 77.37 | 80.83 | 1.50 |  | 83.82 | 0.92 | 81.93 | 83.74 | 87.81 | 1.47 |
| Income Q1 | 73.39 | 1.72 | 68.08 | 73.07 | 78.83 | 2.67 |  | 80.82 | 1.79 | 74.61 | 80.69 | 87.08 | 2.76 |
| Income Q2 | 77.37 | 1.28 | 74.50 | 77.26 | 81.00 | 2.10 |  | 84.17 | 1.14 | 82.23 | 83.99 | 92.09 | 1.60 |
| Income Q3 | 78.10 | 1.21 | 73.25 | 78.09 | 80.92 | 2.05 |  | 84.40 | 1.07 | 82.26 | 84.27 | 88.18 | 1.77 |
| Income Q4 | 78.84 | 1.32 | 74.65 | 78.76 | 83.06 | 2.14 |  | 84.86 | 1.15 | 82.02 | 84.86 | 88.65 | 1.83 |
| Income Q5 | 79.80 | 1.25 | 76.34 | 79.81 | 83.59 | 1.90 |  | 85.59 | 1.23 | 82.57 | 85.45 | 90.24 | 1.79 |
| Q5-Q1 | 6.41 | 1.79 | 2.04 | 6.56 | 11.73 | 3.14 |  | 4.76 | 1.80 | -2.18 | 4.61 | 10.35 | 3.02 |

**Supplementary Table 5. Correlations of the area deprivation index with district-level quality-adjusted life expectancy (QALE) by gender and income quintile, 2008-2014**

|  |  | Total | | | |  | Men | | | |  | Women | | | |
| --- | --- | --- | --- | --- | --- | --- | --- | --- | --- | --- | --- | --- | --- | --- | --- |
|  |  | r | 95%CI(L) | 95%CI(U) | P-value |  | r | 95%CI(L) | 95%CI(U) | P-value |  | r | 95%CI(L) | 95%CI(U) | P-value |
| % of residents living alone | |  |  |  |  |  |  |  |  |  |  |  |  |  |  |
|  | Q1 (Lowest) | -0.57 | -0.65 | -0.48 | <.0001 |  | -0.62 | -0.69 | -0.53 | <.0001 |  | -0.37 | -0.47 | -0.25 | <.0001 |
|  | Q2 | -0.57 | -0.65 | -0.48 | <.0001 |  | -0.59 | -0.66 | -0.50 | <.0001 |  | 0.02 | -0.10 | 0.15 | 0.7174 |
|  | Q3 | -0.34 | -0.44 | -0.22 | <.0001 |  | -0.45 | -0.54 | -0.34 | <.0001 |  | 0.03 | -0.09 | 0.16 | 0.6067 |
|  | Q4 | -0.29 | -0.40 | -0.17 | <.0001 |  | -0.41 | -0.51 | -0.30 | <.0001 |  | 0.05 | -0.08 | 0.17 | 0.481 |
|  | Q5 (Highest) | -0.23 | -0.35 | -0.11 | 0.0002 |  | -0.37 | -0.48 | -0.26 | <.0001 |  | 0.09 | -0.04 | 0.21 | 0.1605 |
| % without housing ownership | |  |  |  |  |  |  |  |  |  |  |  |  |  |  |
|  | Q1 (Lowest) | -0.61 | -0.68 | -0.52 | <.0001 |  | -0.63 | -0.70 | -0.55 | <.0001 |  | -0.44 | -0.54 | -0.33 | <.0001 |
|  | Q2 | -0.61 | -0.68 | -0.52 | <.0001 |  | -0.56 | -0.64 | -0.47 | <.0001 |  | 0.05 | -0.07 | 0.18 | 0.4171 |
|  | Q3 | -0.37 | -0.48 | -0.26 | <.0001 |  | -0.49 | -0.58 | -0.39 | <.0001 |  | 0.03 | -0.10 | 0.15 | 0.6482 |
|  | Q4 | -0.32 | -0.43 | -0.21 | <.0001 |  | -0.48 | -0.57 | -0.38 | <.0001 |  | 0.05 | -0.07 | 0.18 | 0.4102 |
|  | Q5 (Highest) | -0.38 | -0.48 | -0.26 | <.0001 |  | -0.48 | -0.57 | -0.38 | <.0001 |  | -0.01 | -0.13 | 0.12 | 0.9348 |
| % without car ownership | |  |  |  |  |  |  |  |  |  |  |  |  |  |  |
|  | Q1 (Lowest) | -0.43 | -0.53 | -0.33 | <.0001 |  | -0.49 | -0.58 | -0.39 | <.0001 |  | -0.26 | -0.37 | -0.14 | <.0001 |
|  | Q2 | -0.43 | -0.53 | -0.33 | <.0001 |  | -0.45 | -0.55 | -0.35 | <.0001 |  | 0.03 | -0.10 | 0.15 | 0.6608 |
|  | Q3 | -0.25 | -0.37 | -0.13 | <.0001 |  | -0.35 | -0.46 | -0.24 | <.0001 |  | 0.07 | -0.06 | 0.19 | 0.2969 |
|  | Q4 | -0.18 | -0.30 | -0.05 | 0.0049 |  | -0.24 | -0.36 | -0.12 | 0.0001 |  | 0.07 | -0.06 | 0.19 | 0.2731 |
|  | Q5 (Highest) | -0.10 | -0.22 | 0.03 | 0.1246 |  | -0.20 | -0.32 | -0.07 | 0.0017 |  | 0.13 | 0.01 | 0.25 | 0.0392 |
| % with poor housing environment | |  |  |  |  |  |  |  |  |  |  |  |  |  |  |
|  | Q1 (Lowest) | -0.60 | -0.68 | -0.52 | <.0001 |  | -0.65 | -0.72 | -0.57 | <.0001 |  | -0.35 | -0.46 | -0.24 | <.0001 |
|  | Q2 | -0.60 | -0.68 | -0.52 | <.0001 |  | -0.58 | -0.65 | -0.49 | <.0001 |  | 0.02 | -0.11 | 0.14 | 0.7775 |
|  | Q3 | -0.40 | -0.50 | -0.29 | <.0001 |  | -0.47 | -0.57 | -0.37 | <.0001 |  | 0.00 | -0.13 | 0.13 | 0.9993 |
|  | Q4 | -0.34 | -0.44 | -0.22 | <.0001 |  | -0.50 | -0.59 | -0.40 | <.0001 |  | 0.09 | -0.03 | 0.21 | 0.1502 |
|  | Q5 (Highest) | -0.37 | -0.48 | -0.26 | <.0001 |  | -0.47 | -0.56 | -0.37 | <.0001 |  | -0.01 | -0.14 | 0.11 | 0.8268 |
| % of non-apartment housing | |  |  |  |  |  |  |  |  |  |  |  |  |  |  |
|  | Q1 (Lowest) | -0.58 | -0.66 | -0.50 | <.0001 |  | -0.64 | -0.71 | -0.56 | <.0001 |  | -0.35 | -0.45 | -0.23 | <.0001 |
|  | Q2 | -0.58 | -0.66 | -0.50 | <.0001 |  | -0.60 | -0.68 | -0.52 | <.0001 |  | 0.00 | -0.13 | 0.12 | 0.9512 |
|  | Q3 | -0.35 | -0.46 | -0.24 | <.0001 |  | -0.46 | -0.56 | -0.36 | <.0001 |  | 0.07 | -0.05 | 0.20 | 0.2503 |
|  | Q4 | -0.33 | -0.43 | -0.21 | <.0001 |  | -0.43 | -0.53 | -0.32 | <.0001 |  | 0.04 | -0.08 | 0.17 | 0.483 |
|  | Q5 (Highest) | -0.28 | -0.39 | -0.16 | <.0001 |  | -0.39 | -0.49 | -0.28 | <.0001 |  | 0.05 | -0.07 | 0.18 | 0.4073 |
| % with a female household head | |  |  |  |  |  |  |  |  |  |  |  |  |  |  |
|  | Q1 (Lowest) | -0.37 | -0.47 | -0.26 | <.0001 |  | -0.41 | -0.51 | -0.30 | <.0001 |  | -0.26 | -0.37 | -0.14 | <.0001 |
|  | Q2 | -0.37 | -0.47 | -0.26 | <.0001 |  | -0.37 | -0.47 | -0.26 | <.0001 |  | 0.06 | -0.06 | 0.19 | 0.3261 |
|  | Q3 | -0.16 | -0.28 | -0.04 | 0.0117 |  | -0.27 | -0.38 | -0.15 | <.0001 |  | 0.11 | -0.02 | 0.23 | 0.0976 |
|  | Q4 | -0.05 | -0.17 | 0.08 | 0.4816 |  | -0.15 | -0.27 | -0.02 | 0.0218 |  | 0.16 | 0.04 | 0.28 | 0.0114 |
|  | Q5 (Highest) | 0.00 | -0.13 | 0.12 | 0.9513 |  | -0.14 | -0.26 | -0.02 | 0.0272 |  | 0.22 | 0.09 | 0.33 | 0.0007 |
| % with low educational attainment | |  |  |  |  |  |  |  |  |  |  |  |  |  |  |
|  | Q1 (Lowest) | -0.80 | -0.84 | -0.75 | <.0001 |  | -0.84 | -0.87 | -0.80 | <.0001 |  | -0.56 | -0.64 | -0.47 | <.0001 |
|  | Q2 | -0.80 | -0.84 | -0.75 | <.0001 |  | -0.76 | -0.81 | -0.70 | <.0001 |  | -0.06 | -0.18 | 0.07 | 0.3543 |
|  | Q3 | -0.52 | -0.61 | -0.42 | <.0001 |  | -0.64 | -0.71 | -0.55 | <.0001 |  | -0.03 | -0.15 | 0.10 | 0.6653 |
|  | Q4 | -0.48 | -0.57 | -0.37 | <.0001 |  | -0.61 | -0.68 | -0.52 | <.0001 |  | -0.03 | -0.15 | 0.10 | 0.6939 |
|  | Q5 (Highest) | -0.48 | -0.57 | -0.37 | <.0001 |  | -0.60 | -0.67 | -0.51 | <.0001 |  | -0.04 | -0.17 | 0.08 | 0.4959 |
| % of elderly | |  |  |  |  |  |  |  |  |  |  |  |  |  |  |
|  | Q1 (Lowest) | -0.69 | -0.75 | -0.62 | <.0001 |  | -0.73 | -0.78 | -0.67 | <.0001 |  | -0.46 | -0.55 | -0.35 | <.0001 |
|  | Q2 | -0.69 | -0.75 | -0.62 | <.0001 |  | -0.65 | -0.72 | -0.57 | <.0001 |  | 0.08 | -0.04 | 0.20 | 0.2023 |
|  | Q3 | -0.38 | -0.48 | -0.27 | <.0001 |  | -0.51 | -0.60 | -0.41 | <.0001 |  | 0.09 | -0.04 | 0.21 | 0.1685 |
|  | Q4 | -0.32 | -0.43 | -0.20 | <.0001 |  | -0.48 | -0.57 | -0.38 | <.0001 |  | 0.11 | -0.01 | 0.23 | 0.0796 |
|  | Q5 (Highest) | -0.33 | -0.44 | -0.21 | <.0001 |  | -0.44 | -0.54 | -0.34 | <.0001 |  | 0.06 | -0.06 | 0.19 | 0.3218 |
|  |  |  |  |  |  |  |  |  |  |  |  |  |  |  |  |
| % of male unemployment | |  |  |  |  |  |  |  |  |  |  |  |  |  |  |
|  | Q1 (Lowest) | 0.38 | 0.27 | 0.48 | <.0001 |  | 0.41 | 0.30 | 0.51 | <.0001 |  | 0.20 | 0.08 | 0.32 | 0.0014 |
|  | Q2 | 0.38 | 0.27 | 0.48 | <.0001 |  | 0.35 | 0.24 | 0.46 | <.0001 |  | -0.19 | -0.31 | -0.07 | 0.0025 |
|  | Q3 | 0.21 | 0.08 | 0.32 | 0.0011 |  | 0.27 | 0.15 | 0.38 | <.0001 |  | -0.09 | -0.22 | 0.03 | 0.1438 |
|  | Q4 | 0.19 | 0.06 | 0.30 | 0.0033 |  | 0.35 | 0.24 | 0.46 | <.0001 |  | -0.13 | -0.25 | -0.01 | 0.0407 |
|  | Q5 (Highest) | 0.26 | 0.13 | 0.37 | <.0001 |  | 0.32 | 0.20 | 0.43 | <.0001 |  | 0.01 | -0.12 | 0.13 | 0.8819 |
| % of low social class | |  |  |  |  |  |  |  |  |  |  |  |  |  |  |
|  | Q1 (Lowest) | -0.79 | -0.83 | -0.74 | <.0001 |  | -0.81 | -0.85 | -0.76 | <.0001 |  | -0.59 | -0.66 | -0.50 | <.0001 |
|  | Q2 | -0.79 | -0.83 | -0.74 | <.0001 |  | -0.74 | -0.80 | -0.68 | <.0001 |  | -0.07 | -0.19 | 0.06 | 0.3072 |
|  | Q3 | -0.56 | -0.64 | -0.47 | <.0001 |  | -0.66 | -0.72 | -0.58 | <.0001 |  | -0.11 | -0.23 | 0.02 | 0.0886 |
|  | Q4 | -0.53 | -0.61 | -0.43 | <.0001 |  | -0.66 | -0.72 | -0.58 | <.0001 |  | -0.09 | -0.21 | 0.04 | 0.1624 |
|  | Q5 (Highest) | -0.58 | -0.65 | -0.49 | <.0001 |  | -0.66 | -0.73 | -0.59 | <.0001 |  | -0.15 | -0.27 | -0.02 | 0.019 |
| % divorced or separated | |  |  |  |  |  |  |  |  |  |  |  |  |  |  |
|  | Q1 (Lowest) | -0.77 | -0.82 | -0.72 | <.0001 |  | -0.81 | -0.85 | -0.76 | <.0001 |  | -0.55 | -0.64 | -0.46 | <.0001 |
|  | Q2 | -0.77 | -0.82 | -0.72 | <.0001 |  | -0.72 | -0.78 | -0.66 | <.0001 |  | -0.02 | -0.14 | 0.11 | 0.7758 |
|  | Q3 | -0.47 | -0.56 | -0.36 | <.0001 |  | -0.60 | -0.67 | -0.51 | <.0001 |  | 0.01 | -0.11 | 0.14 | 0.8437 |
|  | Q4 | -0.40 | -0.50 | -0.29 | <.0001 |  | -0.55 | -0.63 | -0.46 | <.0001 |  | 0.03 | -0.10 | 0.15 | 0.642 |
|  | Q5 (Highest) | -0.40 | -0.50 | -0.29 | <.0001 |  | -0.53 | -0.62 | -0.44 | <.0001 |  | 0.01 | -0.11 | 0.14 | 0.8256 |
| Area deprivation index | |  |  |  |  |  |  |  |  |  |  |  |  |  |  |
|  | Q1 (Lowest) | -0.76 | -0.81 | -0.70 | <.0001 |  | -0.80 | -0.85 | -0.76 | <.0001 |  | -0.52 | -0.61 | -0.42 | <.0001 |
|  | Q2 | -0.76 | -0.81 | -0.70 | <.0001 |  | -0.73 | -0.78 | -0.67 | <.0001 |  | -0.02 | -0.15 | 0.10 | 0.7378 |
|  | Q3 | -0.47 | -0.56 | -0.37 | <.0001 |  | -0.59 | -0.67 | -0.51 | <.0001 |  | 0.01 | -0.12 | 0.13 | 0.9016 |
|  | Q4 | -0.40 | -0.50 | -0.29 | <.0001 |  | -0.54 | -0.62 | -0.44 | <.0001 |  | 0.03 | -0.10 | 0.15 | 0.6622 |
|  | Q5 (Highest) | -0.39 | -0.49 | -0.27 | <.0001 |  | -0.52 | -0.60 | -0.42 | <.0001 |  | 0.03 | -0.10 | 0.15 | 0.6761 |

**Supplementary Table 6. Correlations of the area deprivation index with district-level life expectancy (LE) by gender and income quintile, 2008-2014**

|  |  | Total | | | |  | Men | | | |  | Women | | | |
| --- | --- | --- | --- | --- | --- | --- | --- | --- | --- | --- | --- | --- | --- | --- | --- |
|  |  | r | 95%CI(L) | 95%CI(U) | P-value |  | r | 95%CI(L) | 95%CI(U) | P-value |  | r | 95%CI(L) | 95%CI(U) | P-value |
| % of residents living alone | |  |  |  |  |  |  |  |  |  |  |  |  |  |  |
|  | Q1 (Lowest) | -0.59 | -0.67 | -0.50 | <.0001 |  | -0.62 | -0.69 | -0.54 | <.0001 |  | -0.45 | -0.54 | -0.34 | <.0001 |
|  | Q2 | -0.48 | -0.57 | -0.38 | <.0001 |  | -0.60 | -0.68 | -0.52 | <.0001 |  | 0.01 | -0.12 | 0.14 | 0.8727 |
|  | Q3 | -0.35 | -0.46 | -0.24 | <.0001 |  | -0.48 | -0.57 | -0.38 | <.0001 |  | 0.02 | -0.11 | 0.15 | 0.7505 |
|  | Q4 | -0.32 | -0.43 | -0.20 | <.0001 |  | -0.48 | -0.57 | -0.38 | <.0001 |  | 0.07 | -0.05 | 0.19 | 0.2683 |
|  | Q5 (Highest) | -0.30 | -0.41 | -0.18 | <.0001 |  | -0.45 | -0.55 | -0.35 | <.0001 |  | 0.03 | -0.10 | 0.15 | 0.6472 |
| % without housing ownership | |  |  |  |  |  |  |  |  |  |  |  |  |  |  |
|  | Q1 (Lowest) | -0.65 | -0.72 | -0.57 | <.0001 |  | -0.65 | -0.72 | -0.57 | <.0001 |  | -0.55 | -0.63 | -0.46 | <.0001 |
|  | Q2 | -0.52 | -0.61 | -0.43 | <.0001 |  | -0.61 | -0.68 | -0.52 | <.0001 |  | -0.06 | -0.18 | 0.07 | 0.3677 |
|  | Q3 | -0.44 | -0.53 | -0.33 | <.0001 |  | -0.56 | -0.64 | -0.47 | <.0001 |  | -0.04 | -0.16 | 0.09 | 0.5563 |
|  | Q4 | -0.37 | -0.47 | -0.26 | <.0001 |  | -0.52 | -0.60 | -0.42 | <.0001 |  | 0.04 | -0.09 | 0.16 | 0.5628 |
|  | Q5 (Highest) | -0.49 | -0.58 | -0.39 | <.0001 |  | -0.60 | -0.68 | -0.52 | <.0001 |  | -0.12 | -0.24 | 0.01 | 0.0716 |
| % without car ownership | |  |  |  |  |  |  |  |  |  |  |  |  |  |  |
|  | Q1 (Lowest) | -0.43 | -0.52 | -0.32 | <.0001 |  | -0.48 | -0.57 | -0.38 | <.0001 |  | -0.28 | -0.39 | -0.16 | <.0001 |
|  | Q2 | -0.35 | -0.46 | -0.24 | <.0001 |  | -0.45 | -0.54 | -0.34 | <.0001 |  | 0.02 | -0.11 | 0.14 | 0.7657 |
|  | Q3 | -0.22 | -0.34 | -0.10 | 0.0005 |  | -0.33 | -0.44 | -0.22 | <.0001 |  | 0.10 | -0.03 | 0.22 | 0.1336 |
|  | Q4 | -0.18 | -0.30 | -0.06 | 0.004 |  | -0.31 | -0.42 | -0.19 | <.0001 |  | 0.09 | -0.03 | 0.22 | 0.1482 |
|  | Q5 (Highest) | -0.13 | -0.25 | -0.01 | 0.0396 |  | -0.29 | -0.40 | -0.17 | <.0001 |  | 0.13 | 0.01 | 0.25 | 0.0394 |
| % with poor housing environment | |  |  |  |  |  |  |  |  |  |  |  |  |  |  |
|  | Q1 (Lowest) | -0.63 | -0.70 | -0.55 | <.0001 |  | -0.66 | -0.73 | -0.58 | <.0001 |  | -0.45 | -0.54 | -0.34 | <.0001 |
|  | Q2 | -0.50 | -0.59 | -0.40 | <.0001 |  | -0.60 | -0.67 | -0.51 | <.0001 |  | 0.03 | -0.10 | 0.15 | 0.6965 |
|  | Q3 | -0.41 | -0.50 | -0.29 | <.0001 |  | -0.52 | -0.60 | -0.42 | <.0001 |  | -0.01 | -0.13 | 0.12 | 0.9343 |
|  | Q4 | -0.34 | -0.45 | -0.23 | <.0001 |  | -0.54 | -0.62 | -0.45 | <.0001 |  | 0.16 | 0.03 | 0.28 | 0.0124 |
|  | Q5 (Highest) | -0.42 | -0.52 | -0.31 | <.0001 |  | -0.53 | -0.62 | -0.44 | <.0001 |  | -0.07 | -0.19 | 0.05 | 0.268 |
| % of non-apartment housing | |  |  |  |  |  |  |  |  |  |  |  |  |  |  |
|  | Q1 (Lowest) | -0.60 | -0.68 | -0.51 | <.0001 |  | -0.64 | -0.71 | -0.56 | <.0001 |  | -0.42 | -0.52 | -0.31 | <.0001 |
|  | Q2 | -0.51 | -0.60 | -0.41 | <.0001 |  | -0.59 | -0.67 | -0.50 | <.0001 |  | 0.00 | -0.13 | 0.13 | 0.9993 |
|  | Q3 | -0.35 | -0.45 | -0.23 | <.0001 |  | -0.49 | -0.58 | -0.39 | <.0001 |  | 0.09 | -0.03 | 0.21 | 0.156 |
|  | Q4 | -0.34 | -0.44 | -0.22 | <.0001 |  | -0.50 | -0.59 | -0.40 | <.0001 |  | 0.09 | -0.04 | 0.21 | 0.1732 |
|  | Q5 (Highest) | -0.31 | -0.42 | -0.20 | <.0001 |  | -0.46 | -0.55 | -0.35 | <.0001 |  | 0.03 | -0.10 | 0.15 | 0.6521 |
| % with a female household head | |  |  |  |  |  |  |  |  |  |  |  |  |  |  |
|  | Q1 (Lowest) | -0.37 | -0.47 | -0.26 | <.0001 |  | -0.40 | -0.50 | -0.29 | <.0001 |  | -0.30 | -0.41 | -0.18 | <.0001 |
|  | Q2 | -0.27 | -0.39 | -0.15 | <.0001 |  | -0.38 | -0.48 | -0.26 | <.0001 |  | 0.01 | -0.12 | 0.14 | 0.8777 |
|  | Q3 | -0.13 | -0.25 | -0.01 | 0.0399 |  | -0.27 | -0.38 | -0.15 | <.0001 |  | 0.14 | 0.01 | 0.26 | 0.0329 |
|  | Q4 | -0.07 | -0.19 | 0.06 | 0.2886 |  | -0.20 | -0.32 | -0.08 | 0.0012 |  | 0.14 | 0.02 | 0.26 | 0.0271 |
|  | Q5 (Highest) | -0.06 | -0.18 | 0.07 | 0.3844 |  | -0.23 | -0.35 | -0.11 | 0.0002 |  | 0.20 | 0.08 | 0.32 | 0.0015 |
| % with low educational attainment | |  |  |  |  |  |  |  |  |  |  |  |  |  |  |
|  | Q1 (Lowest) | -0.82 | -0.86 | -0.78 | <.0001 |  | -0.84 | -0.87 | -0.80 | <.0001 |  | -0.66 | -0.72 | -0.58 | <.0001 |
|  | Q2 | -0.69 | -0.75 | -0.62 | <.0001 |  | -0.78 | -0.83 | -0.73 | <.0001 |  | -0.07 | -0.19 | 0.06 | 0.2737 |
|  | Q3 | -0.56 | -0.64 | -0.47 | <.0001 |  | -0.69 | -0.75 | -0.62 | <.0001 |  | -0.08 | -0.20 | 0.04 | 0.2045 |
|  | Q4 | -0.52 | -0.60 | -0.42 | <.0001 |  | -0.68 | -0.74 | -0.61 | <.0001 |  | -0.01 | -0.14 | 0.11 | 0.8299 |
|  | Q5 (Highest) | -0.56 | -0.64 | -0.47 | <.0001 |  | -0.70 | -0.76 | -0.63 | <.0001 |  | -0.13 | -0.25 | 0.00 | 0.0417 |
| % of elderly | |  |  |  |  |  |  |  |  |  |  |  |  |  |  |
|  | Q1 (Lowest) | -0.72 | -0.77 | -0.65 | <.0001 |  | -0.74 | -0.79 | -0.68 | <.0001 |  | -0.55 | -0.64 | -0.46 | <.0001 |
|  | Q2 | -0.54 | -0.62 | -0.44 | <.0001 |  | -0.66 | -0.73 | -0.58 | <.0001 |  | 0.05 | -0.08 | 0.17 | 0.4417 |
|  | Q3 | -0.40 | -0.50 | -0.28 | <.0001 |  | -0.56 | -0.64 | -0.47 | <.0001 |  | 0.08 | -0.04 | 0.21 | 0.1857 |
|  | Q4 | -0.34 | -0.44 | -0.22 | <.0001 |  | -0.53 | -0.62 | -0.44 | <.0001 |  | 0.15 | 0.02 | 0.27 | 0.0216 |
|  | Q5 (Highest) | -0.40 | -0.50 | -0.29 | <.0001 |  | -0.56 | -0.64 | -0.46 | <.0001 |  | 0.01 | -0.11 | 0.14 | 0.8433 |
|  |  |  |  |  |  |  |  |  |  |  |  |  |  |  |  |
| % of male unemployment | |  |  |  |  |  |  |  |  |  |  |  |  |  |  |
|  | Q1 (Lowest) | 0.41 | 0.30 | 0.51 | <.0001 |  | 0.43 | 0.32 | 0.53 | <.0001 |  | 0.28 | 0.16 | 0.39 | <.0001 |
|  | Q2 | 0.22 | 0.10 | 0.34 | 0.0004 |  | 0.36 | 0.25 | 0.47 | <.0001 |  | -0.25 | -0.36 | -0.12 | <.0001 |
|  | Q3 | 0.20 | 0.08 | 0.32 | 0.0013 |  | 0.33 | 0.21 | 0.44 | <.0001 |  | -0.14 | -0.26 | -0.02 | 0.0272 |
|  | Q4 | 0.16 | 0.04 | 0.28 | 0.0112 |  | 0.35 | 0.23 | 0.45 | <.0001 |  | -0.23 | -0.35 | -0.11 | 0.0003 |
|  | Q5 (Highest) | 0.27 | 0.15 | 0.38 | <.0001 |  | 0.34 | 0.23 | 0.45 | <.0001 |  | 0.02 | -0.11 | 0.14 | 0.8018 |
| % of low social class | |  |  |  |  |  |  |  |  |  |  |  |  |  |  |
|  | Q1 (Lowest) | -0.82 | -0.86 | -0.77 | <.0001 |  | -0.82 | -0.86 | -0.78 | <.0001 |  | -0.69 | -0.75 | -0.62 | <.0001 |
|  | Q2 | -0.71 | -0.77 | -0.64 | <.0001 |  | -0.79 | -0.83 | -0.73 | <.0001 |  | -0.12 | -0.24 | 0.00 | 0.0534 |
|  | Q3 | -0.61 | -0.69 | -0.53 | <.0001 |  | -0.72 | -0.78 | -0.65 | <.0001 |  | -0.18 | -0.29 | -0.05 | 0.0056 |
|  | Q4 | -0.59 | -0.67 | -0.50 | <.0001 |  | -0.74 | -0.79 | -0.68 | <.0001 |  | -0.10 | -0.22 | 0.03 | 0.1228 |
|  | Q5 (Highest) | -0.66 | -0.73 | -0.59 | <.0001 |  | -0.77 | -0.81 | -0.71 | <.0001 |  | -0.25 | -0.37 | -0.13 | <.0001 |
| % divorced or separated | |  |  |  |  |  |  |  |  |  |  |  |  |  |  |
|  | Q1 (Lowest) | -0.80 | -0.84 | -0.75 | <.0001 |  | -0.82 | -0.86 | -0.78 | <.0001 |  | -0.65 | -0.72 | -0.57 | <.0001 |
|  | Q2 | -0.65 | -0.72 | -0.57 | <.0001 |  | -0.76 | -0.81 | -0.70 | <.0001 |  | -0.06 | -0.19 | 0.06 | 0.3378 |
|  | Q3 | -0.49 | -0.58 | -0.39 | <.0001 |  | -0.65 | -0.72 | -0.57 | <.0001 |  | -0.02 | -0.14 | 0.11 | 0.7821 |
|  | Q4 | -0.44 | -0.54 | -0.34 | <.0001 |  | -0.62 | -0.69 | -0.54 | <.0001 |  | 0.01 | -0.11 | 0.14 | 0.8188 |
|  | Q5 (Highest) | -0.49 | -0.58 | -0.39 | <.0001 |  | -0.66 | -0.72 | -0.58 | <.0001 |  | -0.06 | -0.18 | 0.06 | 0.3413 |
| Area deprivation index | |  |  |  |  |  |  |  |  |  |  |  |  |  |  |
|  | Q1 (Lowest) | -0.78 | -0.82 | -0.72 | <.0001 |  | -0.81 | -0.85 | -0.76 | <.0001 |  | -0.61 | -0.68 | -0.52 | <.0001 |
|  | Q2 | -0.64 | -0.71 | -0.56 | <.0001 |  | -0.75 | -0.80 | -0.69 | <.0001 |  | -0.04 | -0.16 | 0.09 | 0.5802 |
|  | Q3 | -0.49 | -0.58 | -0.39 | <.0001 |  | -0.64 | -0.70 | -0.55 | <.0001 |  | -0.01 | -0.14 | 0.11 | 0.8536 |
|  | Q4 | -0.44 | -0.54 | -0.34 | <.0001 |  | -0.62 | -0.69 | -0.54 | <.0001 |  | 0.04 | -0.09 | 0.16 | 0.5419 |
|  | Q5 (Highest) | -0.46 | -0.55 | -0.35 | <.0001 |  | -0.63 | -0.70 | -0.55 | <.0001 |  | -0.04 | -0.16 | 0.09 | 0.5673 |

**Supplementary Table 7. Correlations of district characteristics with district-level quality-adjusted life expectancy (QALE) by gender and income quintile, 2008-2014**

|  |  | Total | | | |  | Men | | | |  | Women | | | |
| --- | --- | --- | --- | --- | --- | --- | --- | --- | --- | --- | --- | --- | --- | --- | --- |
|  |  | r | 95%CI(L) | 95%CI(U) | P-value |  | r | 95%CI(L) | 95%CI(U) | P-value |  | r | 95%CI(L) | 95%CI(U) | P-value |
| Gini index | |  |  |  |  |  |  |  |  |  |  |  |  |  |  |
|  | Q1 (Lowest) | -0.66 | -0.73 | -0.59 | <.0001 |  | -0.69 | -0.75 | -0.62 | <.0001 |  | -0.47 | -0.56 | -0.36 | <.0001 |
|  | Q2 | -0.66 | -0.73 | -0.59 | <.0001 |  | -0.64 | -0.70 | -0.55 | <.0001 |  | 0.02 | -0.10 | 0.15 | 0.7241 |
|  | Q3 | -0.39 | -0.50 | -0.28 | <.0001 |  | -0.49 | -0.58 | -0.39 | <.0001 |  | 0.02 | -0.11 | 0.14 | 0.7554 |
|  | Q4 | -0.33 | -0.44 | -0.22 | <.0001 |  | -0.47 | -0.56 | -0.36 | <.0001 |  | 0.04 | -0.09 | 0.16 | 0.5366 |
|  | Q5 (Highest) | -0.30 | -0.41 | -0.18 | <.0001 |  | -0.43 | -0.53 | -0.32 | <.0001 |  | 0.07 | -0.06 | 0.19 | 0.3055 |
| Social trust | |  |  |  |  |  |  |  |  |  |  |  |  |  |  |
|  | Q1 (Lowest) | -0.57 | -0.65 | -0.48 | <.0001 |  | -0.61 | -0.69 | -0.53 | <.0001 |  | -0.36 | -0.46 | -0.24 | <.0001 |
|  | Q2 | -0.57 | -0.65 | -0.48 | <.0001 |  | -0.49 | -0.58 | -0.39 | <.0001 |  | 0.18 | 0.06 | 0.30 | 0.004 |
|  | Q3 | -0.23 | -0.35 | -0.11 | 0.0003 |  | -0.37 | -0.47 | -0.25 | <.0001 |  | 0.18 | 0.05 | 0.30 | 0.0055 |
|  | Q4 | -0.22 | -0.33 | -0.09 | 0.0006 |  | -0.39 | -0.49 | -0.28 | <.0001 |  | 0.16 | 0.03 | 0.28 | 0.0136 |
|  | Q5 (Highest) | -0.24 | -0.36 | -0.12 | 0.0001 |  | -0.38 | -0.48 | -0.27 | <.0001 |  | 0.10 | -0.02 | 0.23 | 0.1030 |
| Mean height | |  |  |  |  |  |  |  |  |  |  |  |  |  |  |
|  | Q1 (Lowest) | 0.37 | 0.25 | 0.47 | <.0001 |  | 0.34 | 0.22 | 0.44 | <.0001 |  | 0.38 | 0.27 | 0.48 | <.0001 |
|  | Q2 | 0.37 | 0.25 | 0.47 | <.0001 |  | 0.31 | 0.19 | 0.42 | <.0001 |  | 0.29 | 0.17 | 0.40 | <.0001 |
|  | Q3 | 0.35 | 0.24 | 0.46 | <.0001 |  | 0.31 | 0.19 | 0.42 | <.0001 |  | 0.24 | 0.12 | 0.36 | 0.0001 |
|  | Q4 | 0.34 | 0.23 | 0.45 | <.0001 |  | 0.29 | 0.17 | 0.40 | <.0001 |  | 0.24 | 0.12 | 0.35 | 0.0002 |
|  | Q5 (Highest) | 0.36 | 0.25 | 0.47 | <.0001 |  | 0.38 | 0.26 | 0.48 | <.0001 |  | 0.17 | 0.04 | 0.29 | 0.0090 |
|  |  |  |  |  |  |  |  |  |  |  |  |  |  |  |  |
| Current smoking prevalence | |  |  |  |  |  |  |  |  |  |  |  |  |  |  |
|  | Q1 (Lowest) | -0.60 | -0.68 | -0.51 | <.0001 |  | -0.60 | -0.68 | -0.51 | <.0001 |  | -0.48 | -0.57 | -0.38 | <.0001 |
|  | Q2 | -0.60 | -0.68 | -0.51 | <.0001 |  | -0.59 | -0.67 | -0.50 | <.0001 |  | -0.30 | -0.41 | -0.19 | <.0001 |
|  | Q3 | -0.53 | -0.62 | -0.44 | <.0001 |  | -0.52 | -0.61 | -0.43 | <.0001 |  | -0.28 | -0.39 | -0.16 | <.0001 |
|  | Q4 | -0.58 | -0.66 | -0.50 | <.0001 |  | -0.57 | -0.64 | -0.47 | <.0001 |  | -0.33 | -0.43 | -0.21 | <.0001 |
|  | Q5 (Highest) | -0.54 | -0.62 | -0.45 | <.0001 |  | -0.53 | -0.62 | -0.43 | <.0001 |  | -0.29 | -0.40 | -0.17 | <.0001 |
| High-risk drinking prevalence | |  |  |  |  |  |  |  |  |  |  |  |  |  |  |
|  | Q1 (Lowest) | -0.07 | -0.20 | 0.05 | 0.2581 |  | -0.06 | -0.19 | 0.06 | 0.3322 |  | -0.08 | -0.21 | 0.04 | 0.1946 |
|  | Q2 | -0.07 | -0.20 | 0.05 | 0.2581 |  | -0.13 | -0.25 | 0.00 | 0.0443 |  | -0.31 | -0.42 | -0.19 | <.0001 |
|  | Q3 | -0.19 | -0.30 | -0.06 | 0.0034 |  | -0.11 | -0.23 | 0.02 | 0.0948 |  | -0.25 | -0.36 | -0.12 | <.0001 |
|  | Q4 | -0.25 | -0.36 | -0.13 | <.0001 |  | -0.16 | -0.28 | -0.03 | 0.0134 |  | -0.28 | -0.40 | -0.17 | <.0001 |
|  | Q5 (Highest) | -0.20 | -0.32 | -0.08 | 0.0014 |  | -0.13 | -0.25 | -0.01 | 0.0405 |  | -0.27 | -0.38 | -0.15 | <.0001 |
| Exercise rate | |  |  |  |  |  |  |  |  |  |  |  |  |  |  |
|  | Q1 (Lowest) | -0.49 | -0.58 | -0.39 | <.0001 |  | -0.51 | -0.59 | -0.41 | <.0001 |  | -0.35 | -0.46 | -0.24 | <.0001 |
|  | Q2 | -0.49 | -0.58 | -0.39 | <.0001 |  | -0.45 | -0.55 | -0.35 | <.0001 |  | 0.06 | -0.06 | 0.19 | 0.3153 |
|  | Q3 | -0.27 | -0.38 | -0.15 | <.0001 |  | -0.32 | -0.43 | -0.20 | <.0001 |  | 0.00 | -0.13 | 0.12 | 0.9825 |
|  | Q4 | -0.30 | -0.41 | -0.18 | <.0001 |  | -0.42 | -0.52 | -0.31 | <.0001 |  | 0.01 | -0.12 | 0.13 | 0.9363 |
|  | Q5 (Highest) | -0.28 | -0.39 | -0.16 | <.0001 |  | -0.31 | -0.42 | -0.19 | <.0001 |  | -0.09 | -0.21 | 0.04 | 0.1818 |
| BMI ≥ 25 kg/m^2^ | |  |  |  |  |  |  |  |  |  |  |  |  |  |  |
|  | Q1 (Lowest) | -0.42 | -0.52 | -0.31 | <.0001 |  | -0.39 | -0.49 | -0.28 | <.0001 |  | -0.37 | -0.47 | -0.26 | <.0001 |
|  | Q2 | -0.42 | -0.52 | -0.31 | <.0001 |  | -0.38 | -0.48 | -0.26 | <.0001 |  | -0.28 | -0.39 | -0.16 | <.0001 |
|  | Q3 | -0.34 | -0.45 | -0.22 | <.0001 |  | -0.31 | -0.42 | -0.19 | <.0001 |  | -0.17 | -0.29 | -0.05 | 0.0064 |
|  | Q4 | -0.41 | -0.51 | -0.30 | <.0001 |  | -0.35 | -0.46 | -0.23 | <.0001 |  | -0.27 | -0.38 | -0.15 | <.0001 |
|  | Q5 (Highest) | -0.37 | -0.48 | -0.26 | <.0001 |  | -0.33 | -0.44 | -0.22 | <.0001 |  | -0.24 | -0.36 | -0.12 | 0.0001 |
|  |  |  |  |  |  |  |  |  |  |  |  |  |  |  |  |
| % change in population, 2005-2015 | | |  |  |  |  |  |  |  |  |  |  |  |  |  |
|  | Q1 (Lowest) | 0.26 | 0.14 | 0.38 | <.0001 |  | 0.29 | 0.17 | 0.40 | <.0001 |  | 0.19 | 0.06 | 0.30 | 0.0037 |
|  | Q2 | 0.26 | 0.14 | 0.38 | <.0001 |  | 0.27 | 0.15 | 0.38 | <.0001 |  | 0.08 | -0.05 | 0.20 | 0.2395 |
|  | Q3 | 0.12 | -0.01 | 0.24 | 0.0602 |  | 0.21 | 0.09 | 0.33 | 0.0010 |  | -0.04 | -0.16 | 0.09 | 0.5844 |
|  | Q4 | 0.11 | -0.02 | 0.23 | 0.0913 |  | 0.09 | -0.03 | 0.22 | 0.1550 |  | 0.02 | -0.11 | 0.14 | 0.7704 |
|  | Q5 (Highest) | 0.09 | -0.04 | 0.21 | 0.1605 |  | 0.15 | 0.02 | 0.27 | 0.0237 |  | -0.03 | -0.16 | 0.10 | 0.6294 |
|  |  |  |  |  |  |  |  |  |  |  |  |  |  |  |  |
| Number of hospital beds per 1,000 | | |  |  |  |  |  |  |  |  |  |  |  |  |  |
|  | Q1 (Lowest) | -0.24 | -0.35 | -0.12 | 0.0001 |  | -0.22 | -0.34 | -0.10 | 0.0004 |  | -0.24 | -0.36 | -0.12 | 0.0001 |
|  | Q2 | -0.24 | -0.35 | -0.12 | 0.0001 |  | -0.14 | -0.26 | -0.02 | 0.0278 |  | -0.01 | -0.14 | 0.11 | 0.8647 |
|  | Q3 | -0.16 | -0.28 | -0.04 | 0.0096 |  | -0.18 | -0.30 | -0.06 | 0.0041 |  | -0.06 | -0.19 | 0.06 | 0.3296 |
|  | Q4 | -0.14 | -0.26 | -0.01 | 0.0301 |  | -0.15 | -0.27 | -0.02 | 0.0193 |  | -0.06 | -0.18 | 0.07 | 0.3610 |
|  | Q5 (Highest) | -0.11 | -0.23 | 0.01 | 0.0785 |  | -0.15 | -0.27 | -0.02 | 0.0231 |  | 0.00 | -0.12 | 0.13 | 0.9819 |
| Number of doctors per 1,000 | | |  |  |  |  |  |  |  |  |  |  |  |  |  |
|  | Q1 (Lowest) | 0.18 | 0.06 | 0.30 | 0.0035 |  | 0.17 | 0.04 | 0.29 | 0.0077 |  | 0.18 | 0.06 | 0.30 | 0.0041 |
|  | Q2 | 0.18 | 0.06 | 0.30 | 0.0035 |  | 0.17 | 0.05 | 0.29 | 0.0070 |  | 0.09 | -0.03 | 0.22 | 0.1469 |
|  | Q3 | 0.19 | 0.07 | 0.31 | 0.0022 |  | 0.21 | 0.08 | 0.32 | 0.0012 |  | 0.10 | -0.02 | 0.22 | 0.1098 |
|  | Q4 | 0.23 | 0.11 | 0.35 | 0.0003 |  | 0.25 | 0.13 | 0.36 | <.0001 |  | 0.12 | -0.01 | 0.24 | 0.0665 |
|  | Q5 (Highest) | 0.32 | 0.21 | 0.43 | <.0001 |  | 0.29 | 0.17 | 0.40 | <.0001 |  | 0.23 | 0.11 | 0.35 | 0.0002 |

**Supplementary Table 8. Correlations of district characteristics with district-level life expectancy (LE) by gender and income quintile, 2008-2014**

|  |  | Total | | | |  | Men | | | |  | Women | | | |
| --- | --- | --- | --- | --- | --- | --- | --- | --- | --- | --- | --- | --- | --- | --- | --- |
|  |  | r | 95%CI(L) | 95%CI(U) | P-value |  | r | 95%CI(L) | 95%CI(U) | P-value |  | r | 95%CI(L) | 95%CI(U) | P-value |
| Gini index | |  |  |  |  |  |  |  |  |  |  |  |  |  |  |
|  | Q1 (Lowest) | -0.64 | -0.71 | -0.56 | <.0001 |  | -0.68 | -0.74 | -0.61 | <.0001 |  | -0.48 | -0.57 | -0.37 | <.0001 |
|  | Q2 | -0.48 | -0.57 | -0.38 | <.0001 |  | -0.62 | -0.69 | -0.54 | <.0001 |  | 0.08 | -0.05 | 0.20 | 0.2316 |
|  | Q3 | -0.32 | -0.43 | -0.20 | <.0001 |  | -0.50 | -0.59 | -0.40 | <.0001 |  | 0.14 | 0.01 | 0.26 | 0.029 |
|  | Q4 | -0.29 | -0.40 | -0.17 | <.0001 |  | -0.47 | -0.56 | -0.36 | <.0001 |  | 0.13 | 0.00 | 0.25 | 0.0477 |
|  | Q5 (Highest) | -0.32 | -0.42 | -0.20 | <.0001 |  | -0.50 | -0.59 | -0.40 | <.0001 |  | 0.08 | -0.04 | 0.20 | 0.2046 |
| Social trust | |  |  |  |  |  |  |  |  |  |  |  |  |  |  |
|  | Q1 (Lowest) | -0.62 | -0.69 | -0.54 | <.0001 |  | -0.64 | -0.71 | -0.56 | <.0001 |  | -0.49 | -0.58 | -0.38 | <.0001 |
|  | Q2 | -0.39 | -0.49 | -0.28 | <.0001 |  | -0.55 | -0.63 | -0.46 | <.0001 |  | 0.17 | 0.04 | 0.28 | 0.0092 |
|  | Q3 | -0.32 | -0.43 | -0.20 | <.0001 |  | -0.47 | -0.56 | -0.37 | <.0001 |  | 0.10 | -0.03 | 0.22 | 0.1183 |
|  | Q4 | -0.28 | -0.39 | -0.16 | <.0001 |  | -0.47 | -0.56 | -0.36 | <.0001 |  | 0.16 | 0.03 | 0.28 | 0.0142 |
|  | Q5 (Highest) | -0.34 | -0.45 | -0.23 | <.0001 |  | -0.49 | -0.58 | -0.39 | <.0001 |  | 0.02 | -0.11 | 0.14 | 0.8047 |
| Mean height | |  |  |  |  |  |  |  |  |  |  |  |  |  |  |
|  | Q1 (Lowest) | 0.30 | 0.18 | 0.41 | <.0001 |  | 0.27 | 0.15 | 0.38 | <.0001 |  | 0.32 | 0.20 | 0.42 | <.0001 |
|  | Q2 | 0.28 | 0.16 | 0.39 | <.0001 |  | 0.24 | 0.11 | 0.35 | 0.0002 |  | 0.18 | 0.05 | 0.30 | 0.0051 |
|  | Q3 | 0.29 | 0.17 | 0.40 | <.0001 |  | 0.27 | 0.15 | 0.38 | <.0001 |  | 0.22 | 0.09 | 0.33 | 0.0006 |
|  | Q4 | 0.27 | 0.15 | 0.38 | <.0001 |  | 0.28 | 0.16 | 0.39 | <.0001 |  | 0.13 | 0.01 | 0.26 | 0.0346 |
|  | Q5 (Highest) | 0.27 | 0.15 | 0.38 | <.0001 |  | 0.32 | 0.20 | 0.43 | <.0001 |  | 0.06 | -0.06 | 0.19 | 0.3149 |
|  |  |  |  |  |  |  |  |  |  |  |  |  |  |  |  |
| Current smoking prevalence | |  |  |  |  |  |  |  |  |  |  |  |  |  |  |
|  | Q1 (Lowest) | -0.62 | -0.69 | -0.54 | <.0001 |  | -0.61 | -0.68 | -0.52 | <.0001 |  | -0.54 | -0.62 | -0.45 | <.0001 |
|  | Q2 | -0.68 | -0.74 | -0.60 | <.0001 |  | -0.63 | -0.70 | -0.55 | <.0001 |  | -0.32 | -0.43 | -0.20 | <.0001 |
|  | Q3 | -0.59 | -0.66 | -0.50 | <.0001 |  | -0.57 | -0.65 | -0.48 | <.0001 |  | -0.36 | -0.46 | -0.25 | <.0001 |
|  | Q4 | -0.65 | -0.71 | -0.57 | <.0001 |  | -0.63 | -0.70 | -0.55 | <.0001 |  | -0.38 | -0.48 | -0.27 | <.0001 |
|  | Q5 (Highest) | -0.57 | -0.65 | -0.48 | <.0001 |  | -0.57 | -0.65 | -0.48 | <.0001 |  | -0.36 | -0.46 | -0.24 | <.0001 |
| High-risk drinking prevalence | |  |  |  |  |  |  |  |  |  |  |  |  |  |  |
|  | Q1 (Lowest) | -0.03 | -0.16 | 0.09 | 0.6121 |  | -0.05 | -0.17 | 0.08 | 0.4529 |  | 0.01 | -0.12 | 0.13 | 0.8793 |
|  | Q2 | -0.15 | -0.27 | -0.02 | 0.0203 |  | -0.08 | -0.20 | 0.04 | 0.2018 |  | -0.14 | -0.26 | -0.02 | 0.0245 |
|  | Q3 | -0.12 | -0.24 | 0.01 | 0.071 |  | -0.06 | -0.18 | 0.07 | 0.3822 |  | -0.17 | -0.29 | -0.04 | 0.0087 |
|  | Q4 | -0.21 | -0.33 | -0.09 | 0.0007 |  | -0.12 | -0.24 | 0.01 | 0.0649 |  | -0.27 | -0.38 | -0.15 | <.0001 |
|  | Q5 (Highest) | -0.08 | -0.21 | 0.04 | 0.1981 |  | -0.03 | -0.15 | 0.10 | 0.6528 |  | -0.16 | -0.28 | -0.03 | 0.0122 |
| Exercise rate | |  |  |  |  |  |  |  |  |  |  |  |  |  |  |
|  | Q1 (Lowest) | -0.50 | -0.59 | -0.40 | <.0001 |  | -0.52 | -0.60 | -0.42 | <.0001 |  | -0.40 | -0.50 | -0.29 | <.0001 |
|  | Q2 | -0.39 | -0.49 | -0.28 | <.0001 |  | -0.48 | -0.58 | -0.38 | <.0001 |  | 0.03 | -0.10 | 0.15 | 0.6718 |
|  | Q3 | -0.28 | -0.39 | -0.16 | <.0001 |  | -0.39 | -0.49 | -0.28 | <.0001 |  | 0.02 | -0.10 | 0.15 | 0.7357 |
|  | Q4 | -0.27 | -0.38 | -0.15 | <.0001 |  | -0.41 | -0.51 | -0.30 | <.0001 |  | 0.07 | -0.05 | 0.20 | 0.2529 |
|  | Q5 (Highest) | -0.28 | -0.39 | -0.16 | <.0001 |  | -0.38 | -0.48 | -0.27 | <.0001 |  | -0.05 | -0.18 | 0.07 | 0.3912 |
| BMI ≥ 25 kg/m^2^ | |  |  |  |  |  |  |  |  |  |  |  |  |  |  |
|  | Q1 (Lowest) | -0.35 | -0.46 | -0.24 | <.0001 |  | -0.36 | -0.46 | -0.24 | <.0001 |  | -0.27 | -0.38 | -0.15 | <.0001 |
|  | Q2 | -0.34 | -0.44 | -0.22 | <.0001 |  | -0.31 | -0.42 | -0.20 | <.0001 |  | -0.06 | -0.19 | 0.06 | 0.3345 |
|  | Q3 | -0.27 | -0.39 | -0.15 | <.0001 |  | -0.30 | -0.41 | -0.18 | <.0001 |  | -0.08 | -0.21 | 0.04 | 0.186 |
|  | Q4 | -0.32 | -0.43 | -0.21 | <.0001 |  | -0.33 | -0.44 | -0.21 | <.0001 |  | -0.15 | -0.27 | -0.02 | 0.02 |
|  | Q5 (Highest) | -0.25 | -0.36 | -0.13 | <.0001 |  | -0.26 | -0.37 | -0.14 | <.0001 |  | -0.13 | -0.25 | 0.00 | 0.0433 |
|  |  |  |  |  |  |  |  |  |  |  |  |  |  |  |  |
| % change in population, 2005-2015 | | |  |  |  |  |  |  |  |  |  |  |  |  |  |
|  | Q1 (Lowest) | 0.26 | 0.14 | 0.37 | <.0001 |  | 0.28 | 0.16 | 0.40 | <.0001 |  | 0.19 | 0.07 | 0.31 | 0.0026 |
|  | Q2 | 0.23 | 0.10 | 0.34 | 0.0003 |  | 0.26 | 0.14 | 0.37 | <.0001 |  | 0.03 | -0.10 | 0.16 | 0.6422 |
|  | Q3 | 0.14 | 0.01 | 0.26 | 0.0345 |  | 0.16 | 0.04 | 0.28 | 0.0105 |  | 0.02 | -0.11 | 0.14 | 0.7919 |
|  | Q4 | 0.12 | -0.01 | 0.24 | 0.0659 |  | 0.16 | 0.03 | 0.28 | 0.0132 |  | 0.02 | -0.11 | 0.15 | 0.7506 |
|  | Q5 (Highest) | 0.10 | -0.03 | 0.22 | 0.1344 |  | 0.18 | 0.05 | 0.29 | 0.0061 |  | -0.04 | -0.16 | 0.09 | 0.5484 |
|  |  |  |  |  |  |  |  |  |  |  |  |  |  |  |  |
| Number of hospital beds per 1,000 | | |  |  |  |  |  |  |  |  |  |  |  |  |  |
|  | Q1 (Lowest) | -0.29 | -0.40 | -0.17 | <.0001 |  | -0.25 | -0.37 | -0.13 | <.0001 |  | -0.35 | -0.45 | -0.23 | <.0001 |
|  | Q2 | -0.23 | -0.34 | -0.10 | 0.0004 |  | -0.21 | -0.33 | -0.08 | 0.001 |  | -0.17 | -0.29 | -0.04 | 0.0093 |
|  | Q3 | -0.27 | -0.38 | -0.15 | <.0001 |  | -0.26 | -0.37 | -0.14 | <.0001 |  | -0.19 | -0.31 | -0.07 | 0.0023 |
|  | Q4 | -0.19 | -0.31 | -0.07 | 0.0022 |  | -0.20 | -0.32 | -0.08 | 0.0017 |  | -0.12 | -0.25 | 0.00 | 0.0513 |
|  | Q5 (Highest) | -0.18 | -0.30 | -0.06 | 0.0046 |  | -0.22 | -0.34 | -0.10 | 0.0005 |  | -0.05 | -0.18 | 0.07 | 0.4085 |
| Number of doctors per 1,000 | | |  |  |  |  |  |  |  |  |  |  |  |  |  |
|  | Q1 (Lowest) | 0.18 | 0.05 | 0.30 | 0.0053 |  | 0.16 | 0.04 | 0.28 | 0.011 |  | 0.19 | 0.06 | 0.30 | 0.0034 |
|  | Q2 | 0.19 | 0.07 | 0.31 | 0.0025 |  | 0.18 | 0.06 | 0.30 | 0.0037 |  | 0.06 | -0.06 | 0.19 | 0.3143 |
|  | Q3 | 0.22 | 0.10 | 0.34 | 0.0005 |  | 0.23 | 0.11 | 0.35 | 0.0002 |  | 0.12 | 0.00 | 0.24 | 0.0586 |
|  | Q4 | 0.25 | 0.13 | 0.36 | <.0001 |  | 0.26 | 0.14 | 0.37 | <.0001 |  | 0.12 | -0.01 | 0.24 | 0.0657 |
|  | Q5 (Highest) | 0.33 | 0.22 | 0.44 | <.0001 |  | 0.30 | 0.18 | 0.41 | <.0001 |  | 0.27 | 0.15 | 0.38 | <.0001 |

**Supplementary Figure 1. Correlations of quality-adjusted life expectancy (QALE) with inter-quintile income differences in QALE at the district level**

| **Total (r = -0.6073)** | **Men (r = -0.6474)** | **Women (r = -0.2700)** |
| --- | --- | --- |
| (A)  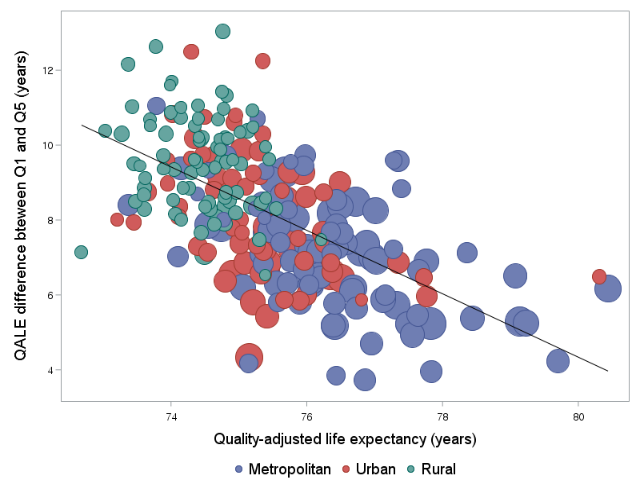 | (B)  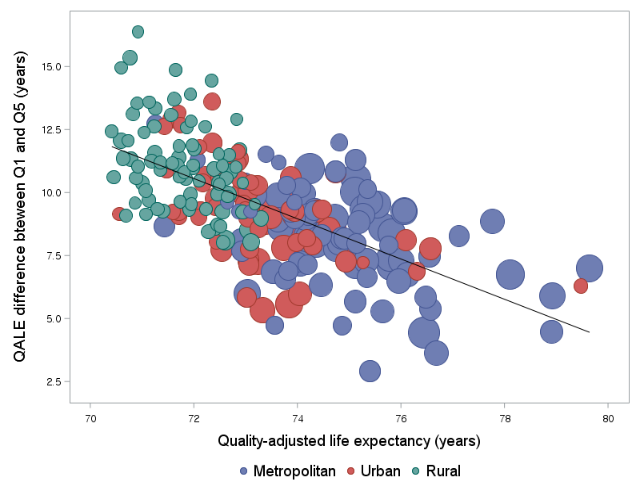 | (C)  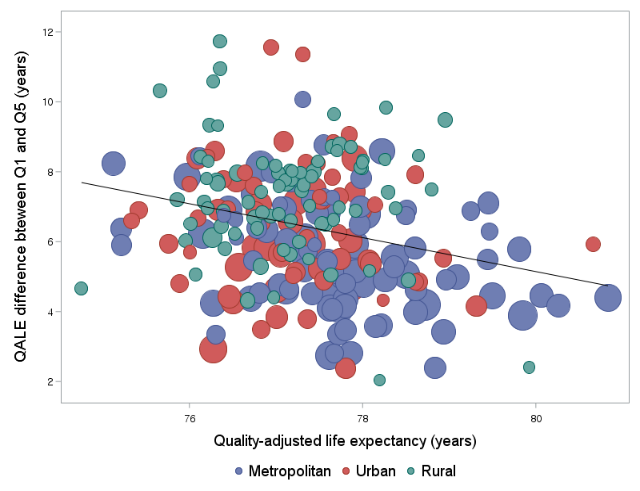 |

**Supplementary Figure 2. Plots of correlations of the area deprivation index with district-level quality-adjusted life expectancy (QALE) by gender and urbanization level**

|  | **Metropolitan** | **Urban** | **Rural** |
| --- | --- | --- | --- |
| **Men** | (A)  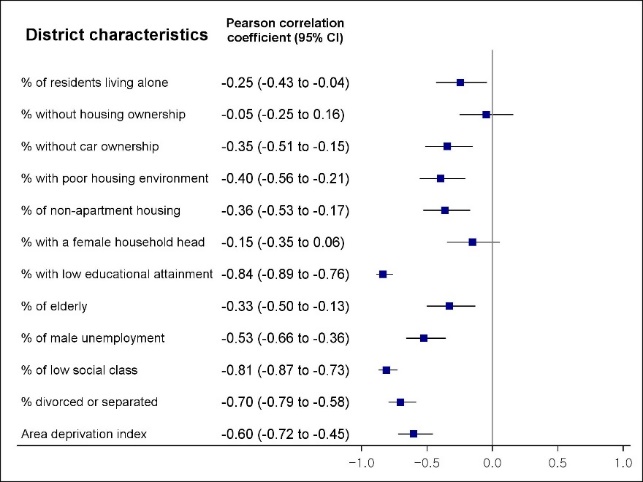 | (B)  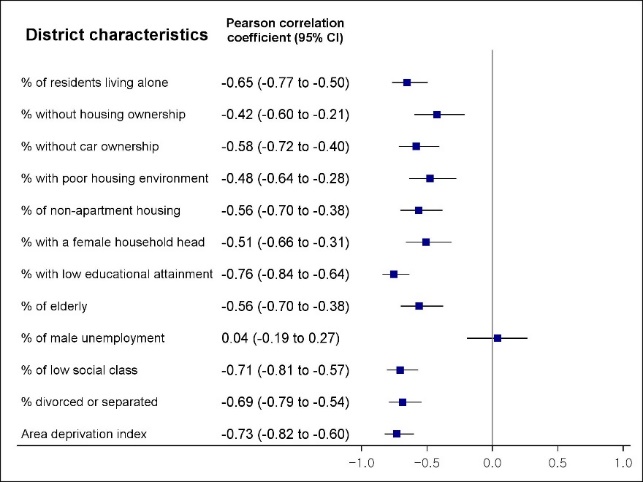 | (C)  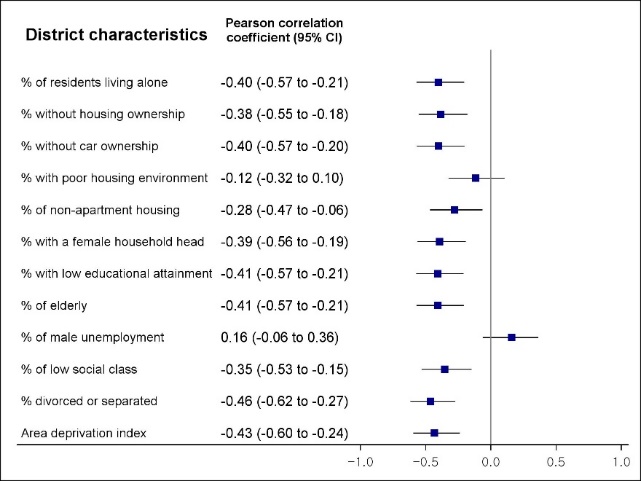 |
| **Women** | (D)  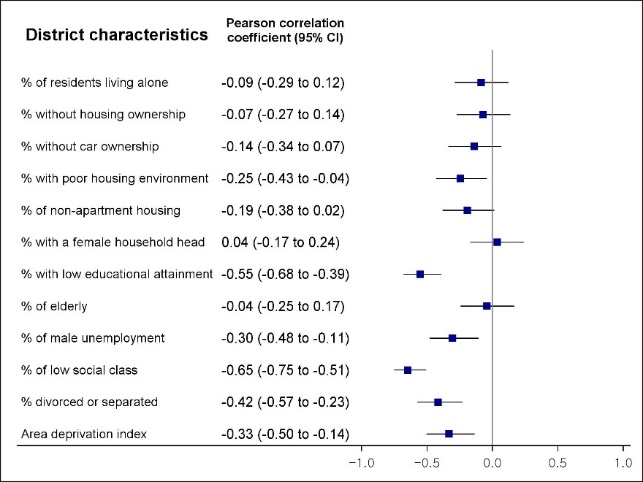 | (E)  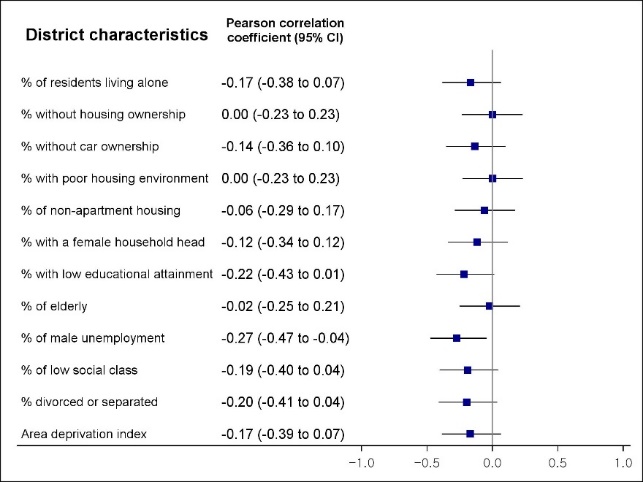 | (F)  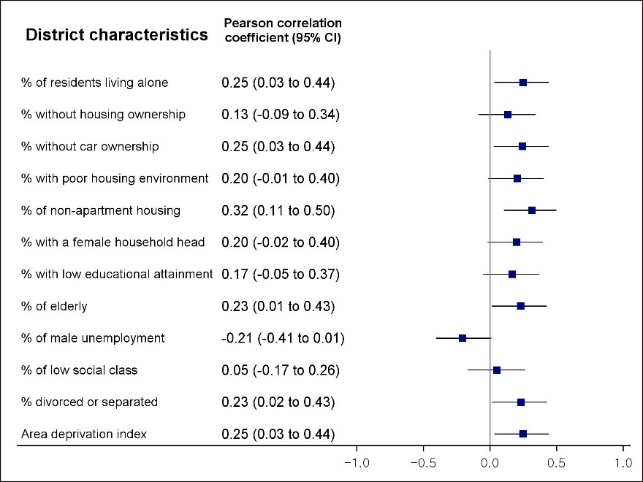 |

**Supplementary Figure 3. Plots of correlations of the area deprivation index with inter-quintile income differences in district-level quality-adjusted life expectancy (QALE) by gender and urbanization level**

|  | Metropolitan | Urban | Rural |
| --- | --- | --- | --- |
| **Men** | (A)  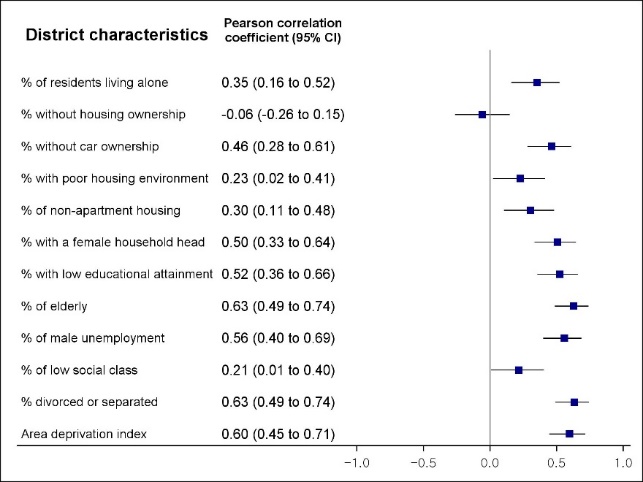 | (B)  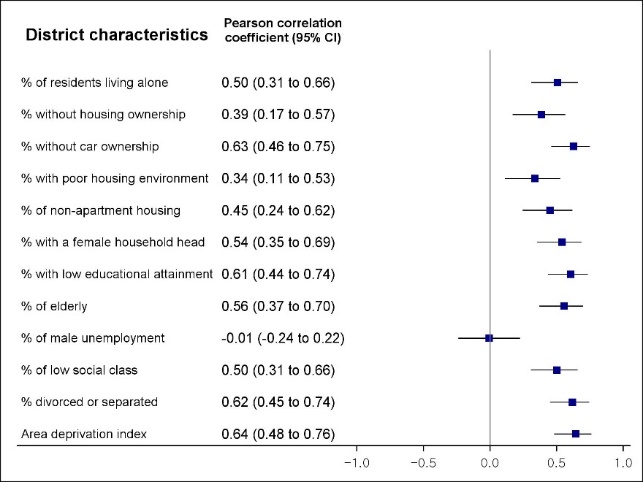 | (C)  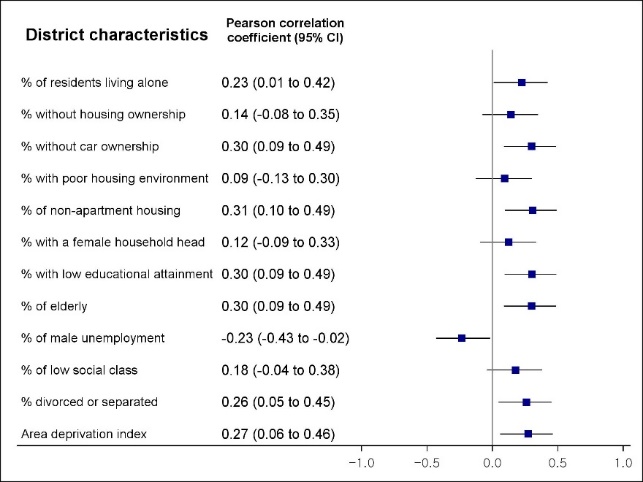 |
| **Women** | (D)  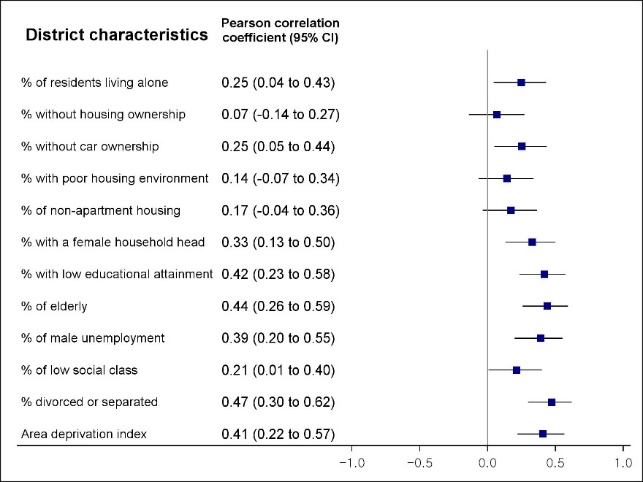 | (E)  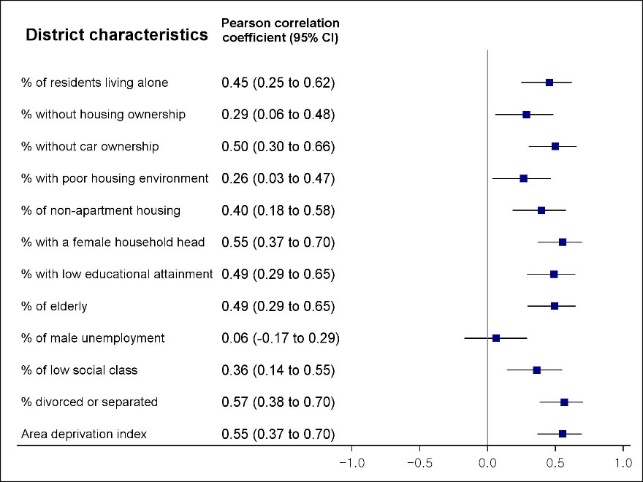 | (F)  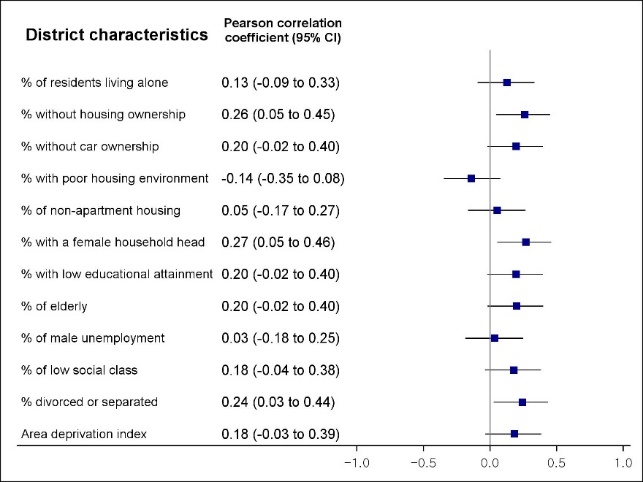 |

**Supplementary Figure 4. Plots of correlations of district characteristics with district-level quality-adjusted life expectancy by gender and urbanization level**

|  | **Metropolitan** | **Urban** | **Rural** |
| --- | --- | --- | --- |
| **Men** | (A)  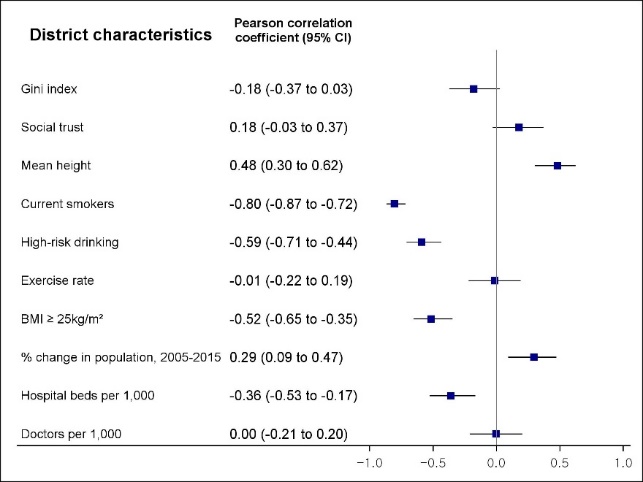 | (B)  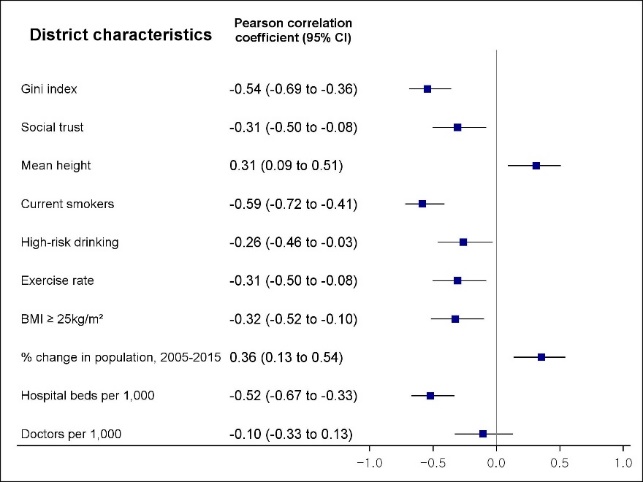 | (C)  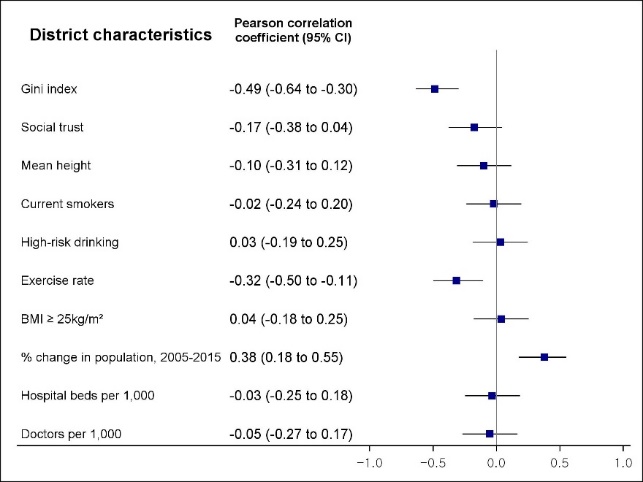 |
| **Women** | (D)  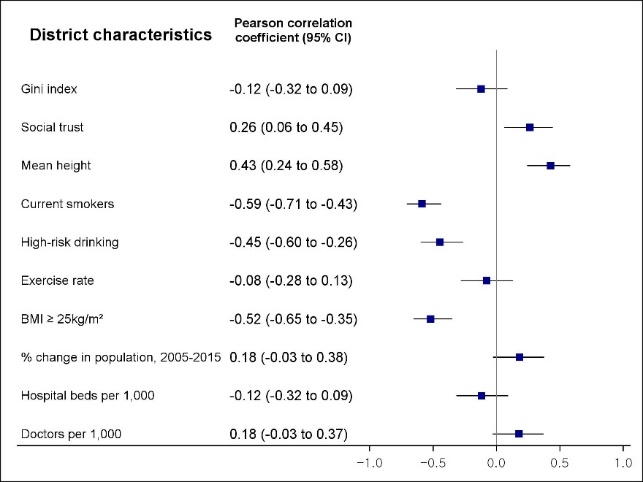 | (E)  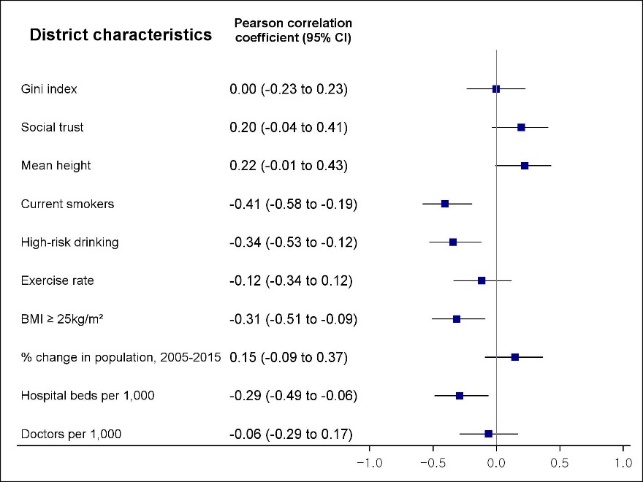 | (F)  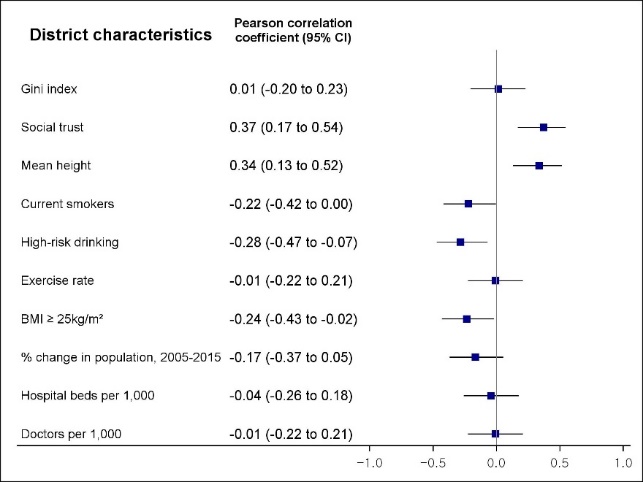 |

**Supplementary Figure 5. Plots of correlations of district characteristics with inter-quintile income differences in district-level quality-adjusted life expectancy by gender and urbanization level**

|  | **Metropolitan** | **Urban** | **Rural** |
| --- | --- | --- | --- |
| **Men** | (A)  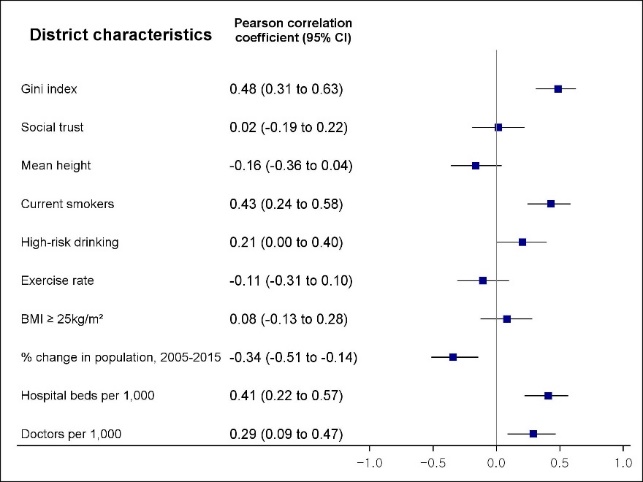 | (B)  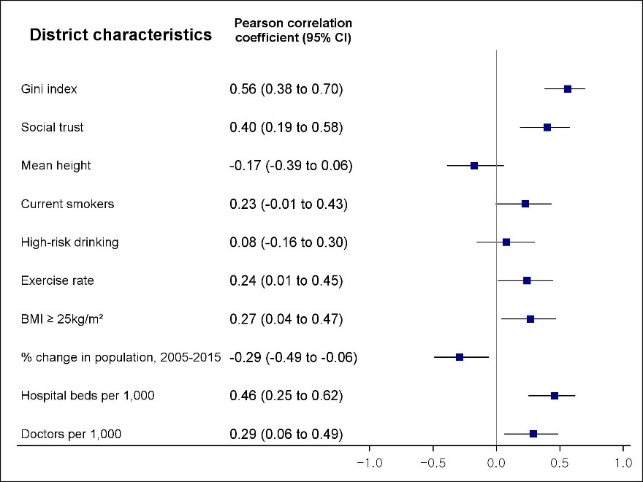 | (C)  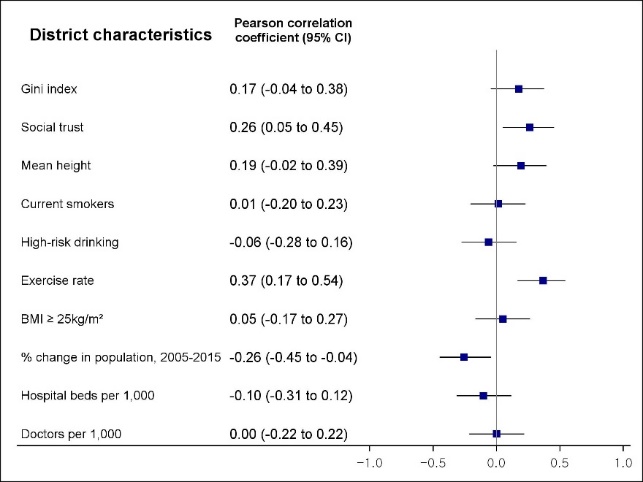 |
| **Women** | (D)  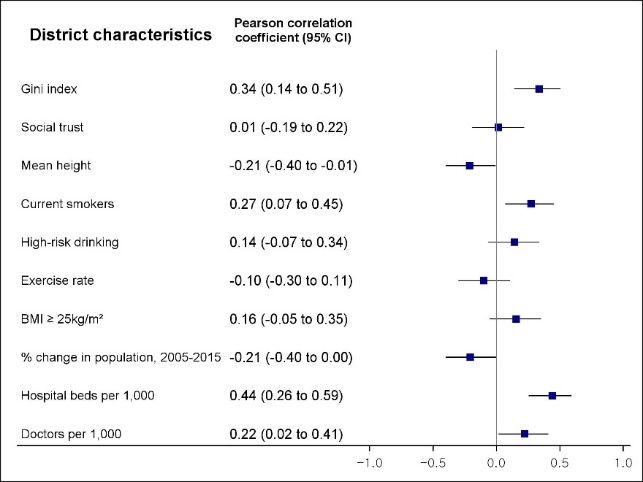 | (E)  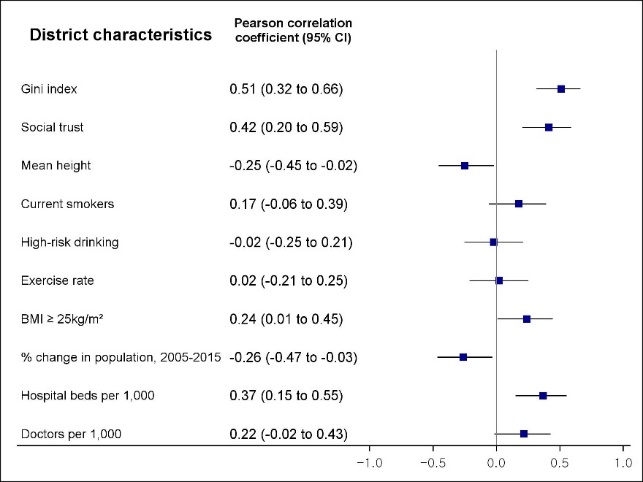 | (F)  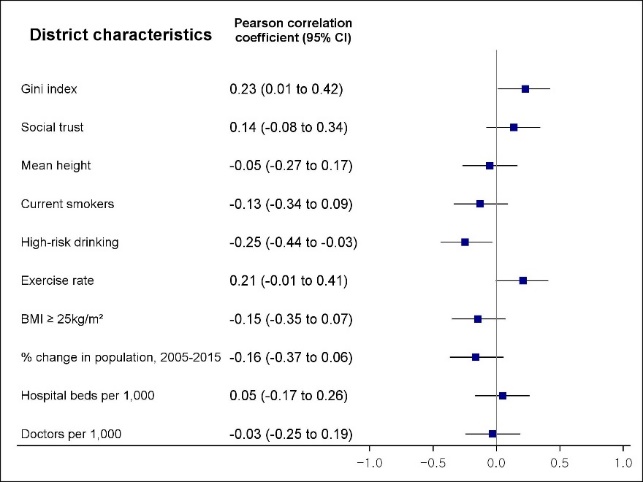 |
